# Single-cell transcriptomics in bone marrow delineates CD56^dim^GranzymeK^+^ subset as intermediate stage in NK cell differentiation

**DOI:** 10.3389/fimmu.2022.1044398

**Published:** 2022-11-24

**Authors:** Janine E. Melsen, Monique M. van Ostaijen-ten Dam, Dorenda J. A. Schoorl, Pieter J. Schol, Daphne A. L. van den Homberg, Arjan C. Lankester, Gertjan Lugthart, Marco W. Schilham

**Affiliations:** Laboratory for Pediatric Immunology, Willem-Alexander Children’s Hospital, Leiden University Medical Center, Leiden, Netherlands

**Keywords:** spectral cytometry, single-cell RNA sequencing, natural killer cells (NK cells), bone marrow, pseudotime analysis

## Abstract

Human natural killer (NK) cells in lymphoid tissues can be categorized into three subsets: CD56^bright^CD16^+^, CD56^dim^CD16^+^ and CD69^+^CXCR6^+^ lymphoid tissue-resident (lt)NK cells. How the three subsets are functionally and developmentally related is currently unknown. Therefore, we performed single-cell RNA sequencing combined with oligonucleotide-conjugated antibodies against CD56, CXCR6, CD117 and CD34 on fresh bone marrow NK cells. A minor CD56^dim^GzmK^+^ subset was identified that shared features with CD56^bright^ and CD56^dim^GzmK^-^ NK cells based on transcriptome, phenotype (NKG2A^high^CD16^low^KLRG1^high^TIGIT^high^) and functional analysis in bone marrow and blood, supportive for an intermediate subset. Pseudotime analysis positioned CD56^bright^, CD56^dim^GzmK^+^ and CD56^dim^GzmK^-^ cells in one differentiation trajectory, while ltNK cells were developmentally separated. Integrative analysis with bone marrow cells from the Human Cell Atlas did not demonstrate a developmental connection between CD34^+^ progenitor and NK cells, suggesting absence of early NK cell stages in bone marrow. In conclusion, single-cell transcriptomics provide new insights on development and differentiation of human NK cells.

## Introduction

Natural killer (NK) cells are innate immune cells, known for their cytotoxicity and effector molecule production upon target cell recognition and/or stimulation by interleukins (1–4). NK cells can be discriminated from the non-cytotoxic innate lymphoid cells (ILCs) based on expression of perforin and Eomes (5–7). Human NK cells in peripheral blood are categorized into two subsets: CD56^bright^CD16^+/-^ and CD56^dim^CD16^+^. In several lymphoid (bone marrow (4, 8), lymph node (4, 8), spleen (4, 8), tonsil (9)) and non-lymphoid tissues (lung (8, 10), intestines (8), uterus (11, 12), liver (13–18)) a third NK cell subset has been described: tissue-resident NK cells (19). The lymphoid tissue-resident (lt)NK cells, and liver-resident NK cells are characterized by the combined expression of CD69 and CXCR6, and absence of the integrin CD49e (4, 20). The integrins CD103 and CD49a are exclusively expressed by mucosal tissue-resident NK cells (8–10, 12).

The functional capacity of the CD56^bright^ and CD56^dim^ NK cells is dependent on the type of stimulation. CD56^dim^ NK cells are potent cytokine and chemokine producers and become highly cytotoxic upon target cell stimulation (1, 2) In contrast, the CD56^bright^ NK cells require interleukin activation to kill target cells and produce cytokines (21). Although, ltNK cells have the highest mRNA expression of *IFNG*, *CCL3*, *CCL4*, *CCL5*, *XCL1* and *XCL2* compared to both CD56^bright^ and CD56^dim^ NK cells as studied by bulk RNA sequencing, standard NK cell function assays (PMA/ionomycin, interleukin or K562 stimulation) did not reveal abundant production of IFN-γ at protein level (4, 20). Moreover, they are less cytotoxic compared to CD56^dim^ NK cells. Heterogeneity within the ltNK cell population or requirement of yet unidentified stimuli might explain the unresponsiveness of the majority of ltNK cells.

It is assumed that the CD56^bright^ NK cells are the precursors of the CD56^dim^ NK cells based on telomere length (22), reconstitution following hematopoietic stem cell transplantation (23) and *in vitro* differentiation studies (22, 24, 25). Following *in vitro* differentiation with cytokines a gain of CD16, killer-cell immunoglobulin-like receptors (KIRs), and loss of IL-7Rα (CD127), CD117, CXCR3, and CCR7 was observed (22, 24). However, whether a cytokine-induced effect *in vitro* is representative for biological differentiation is questionable. The CD56^bright^CD16^+^, CD56^bright^CD27^-^, CD56^dim^CD94^high^, CD56^dim^CD62L^+^ and CD56^dim^CD16^dim^ populations have been independently proposed to represent the intermediate subset based on phenotype and function (24, 26–29). Nevertheless, the detailed sequential steps during differentiation and overlap of these populations remain unclear. In addition, the developmental relation between the ltNK cells, CD56^bright^ and CD56^dim^ NK cells is still unknown. Hypothetically, ltNK cells could either constitute a separate lineage, or could be developed from either the CD56^bright^ or CD56^dim^ NK cells.

Bulk RNA sequencing does not unveil the heterogeneity within the NK cell populations, and therefore single-cell RNA sequencing provides a valuable tool to study both functional characteristics and development of the CD56^bright^, CD56^dim^ and ltNK cells. Multiple single-cell RNA sequencing datasets on blood (30–34) and bone marrow (31, 35) NK cells have been published (36). However, the mRNA expression of some important markers used to identify the known NK cell populations by cytometry is around the lower level of detection for this technology. Therefore, we applied the CITE-seq technology, using oligonucleotide-conjugated antibodies for CD56, CXCR6, CD117 and CD34. We demonstrate considerable heterogeneity within the CD56^dim^ population, and identify a CD56^dim^GzmK^+^ NK cell subset as intermediate differentiation stage between CD56^bright^ and CD56^dim^GzmK^-^GzmB^+^ NK cells. In addition, we propose that resident ltNK cells develop independently from the circulating NK cells and found no evidence of NK cell development in the human bone marrow.

## Materials and methods

### Ethics statement

With approval of the Institutional Review Board (protocols P00.068, P01.028, B17.001 and LUMC healthy voluntary donor service (LuVDS)) and informed consent, blood (fresh or frozen) and bone marrow (fresh or frozen) from healthy controls and one hematopoietic stem cell transplant recipient were analyzed. Fresh blood (n=6) has been collected by the LuVDS, coordinated by the central biobanking facility at the LUMC. Residual fresh bone marrow from one healthy donor was used for single-cell RNA sequencing. Residual splenic tissue from Dutch solid organ transplant donors were used anonymously, in accordance with the Dutch law for organ donation. Tonsils and omental lymph nodes were collected as leftover material from tonsillectomy and bariatric surgery, respectively. Tonsils were removed in the absence of active infection. Lymph nodes were removed only in steady conditions. Results were evaluated anonymously in accordance with Dutch national ethical and professional guidelines (http://www.federa.org).

### Preparation of NK-enriched bone marrow cells

Bone marrow mononuclear cells (BMMC) were isolated by Ficoll density gradient centrifugation (LUMC Pharmacy, Leiden, The Netherlands). Untouched NK cell enrichment was performed by using Mojosort magnetic cell separation (Biolegend, San Diego, CA, USA), according to manufacturer’s instructions. Anti-CD34 was not included in this kit, enabling enrichment of NK progenitor cells. Purity of the population was assessed by flow cytometry (**Figure S1A**). The antibodies used are listed in **Table S1**. Enriched cells were incubated with Fc block (eBioscience, San Diego, CA USA), after which cells were labeled with 1µg/ml oligonucleotide conjugated antibodies specific for CD34, CD56, CD117 and CXCR6 (TotalSeq-A, Biolegend, **Table S2**). The labeling was confirmed on a fraction of the cells by a secondary staining with goat-anti-mouse APC (Becton Dickinson (BD), Franklin Lakes, NJ, USA) (**Figure S1A**). Data was acquired on a LSR-II flow cytometer (BD).

### Single-cell RNA sequencing data acquisition

The NK-enriched cell suspension was loaded on a Chromium Single Cell Chip (10x Genomics, Pleasanton, CA, USA) to encapsulate 10,000 cells with barcoded beads. Library preparation of the mRNA and cell surface bounded antibodies was performed by using the Single Cell 3’ solution v3 (10x Genomics), according to manufacturer’s instructions. The libraries were pooled, and sequencing was performed on one lane of the Illumina NovaSeq 6000 system (Illumina, San Diego, CA, USA).

### Single-cell RNA sequencing data analysis

Cell ranger (software v3.0.2, 10x Genomics) was used to align the sequences to the human genome (hg38) and the antibodies. Barcodes associated with cells were selected based on the distribution of barcode counts and number of UMI counts mapped to each barcode (knee plot). The results from a total of 7000 cells were exported for further analysis in R (v4.0, R Foundation for Statistical Computing, Vienna, Austria) using the Bioconductor workflow as guide (37). The DropletUtils package (38) was used to import the sequence data as SingleCellExperiment. Quality control was performed by using the Scater package (39). Low-quality cells (n=21) with <1000 expressed genes, >8300 expressed genes and >12,5% mitochondrial RNA, were removed (**Figure S1B**). 454 remaining doublets were removed that clustered based on high mRNA and gene content. In total, 6525 cells, 33538 genes and 4 antibodies were included in subsequent analyses. Normalization of the antibody data was performed by using the centered log ratio, as implemented by Seurat (40). The gene expression data was log-transformed and normalized by using deconvolution size factors, as implemented by Scran. The top 2000 most variable genes were selected by computing the variance of the log-counts and fitting a trend to the variance with respect to abundance across the genes (Scran) (**Figure S1C**). Principal component analysis (PCA) was applied and the top 20 PCs were retained (**Figure S1D**). Graph-based infomap clustering (k=30, type=rank) was performed by Igraph (41). To visualize the clusters and expression data, a UMAP embedding was calculated (n_neighbors=30, min.dist=0.6, unless stated otherwise). Heatmaps were generated using the pheatmap package (42). The cell-cycle score was calculated using the cyclone function in Scran. Subclustering of populations was performed by recalculating variable features and principle components. Pseudotime analysis was performed using Slingshot (omega=0.9) (43) and RNA velocity (stochastic model) using scvelo (44, 45) as implemented by velicoraptor (46) based on 50 PCs. The starting clusters were manually determined after lineage identification by Slingshot. To determine which genes change their expression over pseudotime a negative binomial general additive model (GAM) was fitted using the Tradeseq package (47).

### Integration of public single-cell RNA sequence NK cell datasets

The single-cell RNA sequencing datasets (GSE130430) of Yang et al. (31) containing 9367 cells were aligned and aggregated (without normalization) using Cell ranger (v3.1.0). Cells that expressed less than 200 genes or more than 6000 genes were removed. In addition, cells containing less than 10% ribosomal protein coding genes or more than 10% mitochondrial genes were filtered out. The single-cell RNA sequencing datasets (GSE159624) of Crinier et al. (35) containing 28238 cells were aligned and aggregated (without normalization) using Cell ranger (v3.1.0). For each individual donor, cells that expressed more or less than 3 median absolute deviations (MADs) from the median log2 transformed UMI count, more than 3 MADs from the median percentage of mitochondrial genes or less than 10% ribosomal protein coding genes were removed. The Human Cell Atlas (HCA) bone marrow dataset (48) containing 378.000 cells was downloaded using the HCAData package (49). For each individual donor, cells containing less than 3 MADs of median log2 transformed total UMI or gene count, and/or more than 3 MADs of the median percentage of mitochondrial genes were removed. All the files were log transformed and normalized using deconvolution size factors.

Integration of our dataset with the Crinier, Yang and HCA dataset required correction of sequencing depth by recomputing log-normalized expression values after adjusting the size factors [multiBatchNorm function in Batchelor (50)]. Integration of the Crinier, Yang and HCA donors was performed using mutual nearest neighbors (MNN) correction (50), based on the top 2000 most variable genes. Graph-based walktrap clustering was performed using kmeans in Scran. Subclustering was performed with infomap clustering. Both the clustering and the UMAP were based on the corrected PC scores. Reference-based analysis, using the Blueprint (51) and ENCODE (52) datasets, or our own annotated dataset was performed to annotate cell clusters by SingleR (53).

### NK cell stimulation

Peripheral blood mononuclear cells (PBMC) and BMMC were thawed in AIMV-medium (Thermo Fisher Scientific, Waltham, MA, USA) supplemented with 20% Fetal Calf serum (FCS, Sigma-Aldrich, Saint Louis, MI, USA) and 1600 IU/ml DNAse (Merck, Darmstadt, Germany). Alternatively, NK cells were enriched from fresh PBMC or BMMC by Mojosort (Biolegend) or EasySep (StemCell Technologies, Vancouver, Canada) magnetic cell separation according to manufacturer’s instructions. To determine chemokine and cytokine production, cells were cultured in AIM-V medium supplemented with 10% FCS and 1% Penicillin/Streptomycin in the presence of 10 ng/ml IL-12 (PeproTech, Rocky Hill, NJ, USA), 10 ng/ml IL-15 (CellGenix, Freiburg, Germany) and 20 ng/ml IL-18 (20 ng/ml, MBL International, Woburn, MA, USA) for 4h, or K562 cells (overnight) at 37°C. For the CD16 and NKp46/2B4 stimulation, a flat-bottom plate was coated with 5 ug/ml goat anti-mouse antibody (BD) and subsequently with 1 ug/ml anti-CD16 (clone 3G8, Biolegend), or a combination of anti-NKp46 (clone 9E2, BD) and anti-2B4 (clone C1.7, eBioscience) in PBS. 1 ug/ml mouse IgG1 isotype (Biolegend) served as negative control. Cells were transferred to the flat-bottom plate and cultured for 4h (CD16) or overnight (NKp46+2B4) at 37°C. Golgistop (BD) was added after 1h of culture. To assess granzyme K and granzyme B production, fresh PBMC and thawed BMMC were cultured overnight in AIM-V medium supplemented with 10% FCS and 1% Penicillin/Streptomycin in the presence of 10 ng/ml IL-12 and 10 ng/ml IL-15.

### Spleen, tonsil and lymph node cell isolation

Splenic tissues, lymph nodes and tonsils were stored in University of Wisconsin solution at 4˚C and processed within 12h after surgery. Tissues were dispersed through a 70-mm cell strainer. Mononuclear cells were isolated from spleens and tonsils by ficoll density gradient centrifugation. Samples were analyzed immediately by conventional flow cytometry.

### Conventional and spectral flow cytometry

Mononuclear cells were stained with fluorochrome-conjugated antibodies (**Table S1**) in PBS supplemented with 0.5% Bovine Serum Albumin, 2mM EDTA (Merck) and 0.02% NaN_3_ (LUMC Pharmacy), for 30 minutes at room temperature (RT). For spectral cytometry, Brilliant Stain buffer plus (BD) was added to this mix. For the intracellular staining, cells were fixed in 4% paraformaldehyde and permeabilized in 0.1% saponin, as previously described (54). Next, cells were incubated in Fc block (eBioscience) for 10 minutes at RT. Intracellular staining with antibodies (**Table S1**) was performed for 30 minutes at 4°C. For the XCL1 staining, 6.7% donkey serum (Jackson ImmunoResearch, West Grove, PA, USA) was added together with unconjugated polyclonal goat anti-XCL1 to the mix. Finally, cells were incubated with donkey-anti-goat as secondary antibody to detect XCL1 for 30 minutes at 4°C. A reliable bi-modal population was observed upon 4h PMA (12.5 ng/ml) and ionomycin (1 μg/ml) stimulation. For cells only stained extracellularly, DAPI was added prior to measurement to detect dead cells. Data was acquired on a LSRII flow cytometer (BD) or 3L/5L Aurora spectral cytometer (Cytek Biosciences, Fremont, CA, USA), using Diva software (v8.0) or SpectroFlo software (v2.2.3, Cytek), respectively.

### Cytometry data analysis

All cytometry data were analyzed on the OMIQ data science platform (Omiq, Inc, Santa Clara, CA, USA). For spectral cytometry data, FlowAi (55) was applied to detect anomalous events, based on changes in flow rate, or outlier events. Data were compensated, arcsinh transformed, and gated to remove dead cells and doublets, as previously described (56). To remove inter-experiment variation, data was normalized using fda normalization (57). UMAP was applied for visualization (58). NK cell and other subsets were either selected by gating on a 2-dimensional plot, or on the UMAP.

### Statistics

Differentially expressed genes between clusters and subclusters derived from the single-cell RNA sequence data were determined by performing a paired one-sided and two-sided Wilcoxon test, respectively. Each cluster was compared to each of the other clusters. Genes were ranked based on significance. The Wald test was applied, using the associationTest function in Tradeseq (default parameters), to find significant genes for each pseudotime lineage. Statistical testing for differences in phenotype between NK cell subsets was performed using a repeated measures one-way ANOVA (Dunnett or Šidák correction was applied for multiple comparisons) or paired Wilcoxon test. Statistical differences in chemokine production between stimulated and unstimulated NK cell subsets were determined using a paired Friedman test (Dunn’s correction was applied for multiple comparisons). A false discovery rate or (adjusted) P-value <0.05 were considered as statistically significant.

## Results

In this study we performed single-cell RNA sequencing on fresh bone marrow-derived NK and progenitor cells to study function and development of the CD56^bright^, CD56^dim^ and ltNK cells. NK and progenitor cells were negatively enriched from one human bone marrow sample and labeled with oligonucleotide-conjugated antibodies against CD56, CXCR6, CD117 and CD34, which allows identification of subsets at protein level (**Figure S1A**). 475 out of 7000 cells did not fulfill the strict quality control criteria. In total 6525 cells, 33538 genes and 4 antibodies were further analyzed (**Figure S1B**). With a median of 1615 genes and 3687 unique molecular identifiers (UMIs) per cell, a high-resolution single-cell dataset was created. Principal component analysis (PCA) was performed on the top 2000 most variable genes, and the first 20 PCs were used for clustering and uniform manifold approximation and projection (UMAP; **Figures S1C, D**).

### Clustering results in identification of CD34^+^ progenitor cells, ILCs and six distinct NK cell subsets

**Figure 1:**
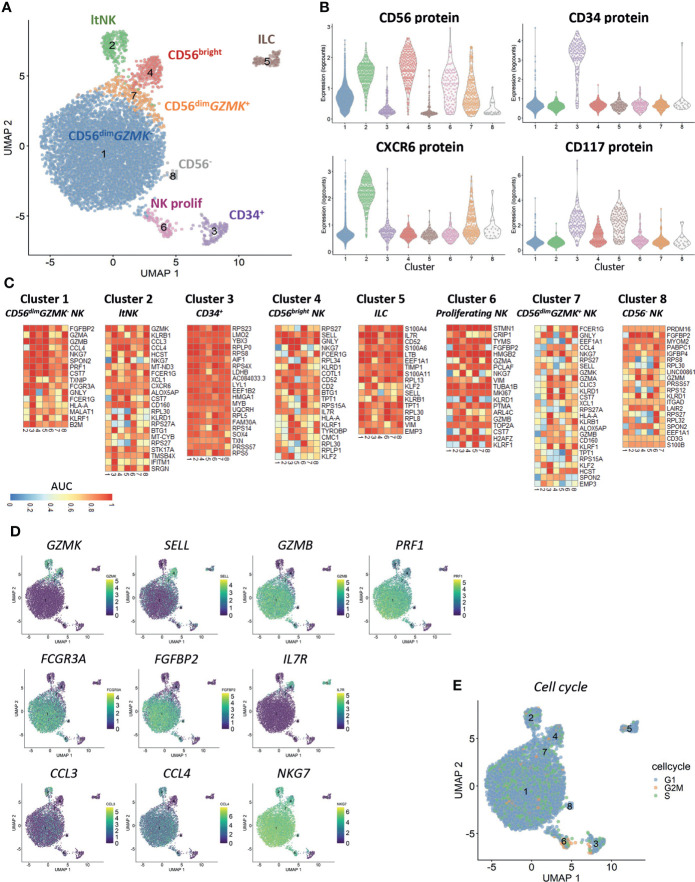
Clustering reveals six distinct NK cell populations. Single-cell RNA sequencing was performed on NK cell-enriched bone marrow cells of one healthy donor. In total 6525 cells, 33538 genes and 4 antibodies were analyzed. A median of 1615 genes per cell was detected. **(A)** The UMAP is based on the top 20 principal components. Clustering on the same components revealed eight distinct clusters (6 NK cell clusters, CD34^+^ cells and innate lymphoid cells (ILCs)). ltNK = lymphoid tissue NK, NK prolif = proliferating NK. **(B)** The protein expression per cluster as determined by oligonucleotide-conjugated antibodies. **(C)** For each cluster a pairwise comparison was performed with each of the 7 other individual clusters. The top 5 upregulated genes for each cluster comparison were determined based on significance by the Wilcoxon test. A maximum of 35 genes is depicted for each cluster (Total = 7 comparisons x 5 genes minus overlapping genes). An AUC >0.5 is considered as upregulated, while an AUC <0.5 is considered as downregulated. **(D)** For a selection of genes, the expression levels are projected on the UMAP plot. **(E)** The cell cycle stage was calculated for each cell based on a list of cell-cycle related genes.

Clustering of cells based on the gene expression revealed the presence of eight different clusters (**Figure 1A**). Based on the CD56 and CXCR6 protein expression, cluster 1 (83% of all cells) and 7 (3.4%) represented CD56^dim^ NK cells, cluster 2 (4%) represented the CXCR6^+^ lymphoid tissue-resident (lt)NK cells and cluster 4 (3.5%) represented the CD56^bright^ NK cells (**Figure 1B**). Cluster 6 (1.6%) was a mixed population of CD56^bright^ and CD56^dim^ NK cells. CD34 was exclusively expressed on cluster 3 (1.9%). CD117 was expressed on CD56^bright^ and CD34^+^ cells (cluster 4 and 3) as well as on CD56^-^CD34^-^ cells in cluster 5 (1.7%). The cells in cluster 8 (0.3%) did not express any of the four proteins.

Based on the differentially expressed genes between clusters, we concluded that CD56^dim^ NK cells were separated into a *GZMK* ^-^ (cluster 1) and *GZMK* ^+^ (cluster 7) subset (**Figures 1C, D**). The CD56^dim^ *GZMK* ^+^ NK cells were further characterized by higher expression of *SELL* (CD62L), and lower expression of *GZMB*, *PRF1* and *FCGR3A* (CD16) and *FGFBP2* compared to CD56^dim^ *GZMK^-^* cells, suggesting that these cells represent an intermediate stage between CD56^bright^ and CD56^dim^ NK cells (**Figures 1C, D**). The CD56^bright^ NK cells in cluster 4 had high expression of markers which are known to be expressed at protein level as well: *SELL*, *CD2* and *IL7R* (**Figures 1C, D**) (19). Among the upregulated genes in the ltNK cells in cluster 2, various chemokines (*CCL3*, *CCL4*, *XCL1*) were included, but also *GZMK* and *CD160* (**Figures 1C, D**). The absence of *NKG7*, and high expression of *IL7R* in cluster 5 suggests that these cells are non-cytotoxic innate lymphoid cells (ILCs, **Figures 1C, D**) (59). Cluster 6 was characterized by upregulation of cell-cycle related genes as also demonstrated by the cell cycle score, thus representing proliferating NK cells (**Figures 1C, E**). The CD56^-^ cells in cluster 8 did express *NKG7* and *FCGR3A*, but had low expression of *PRF1* and *GZMB*, and likely represent CD56^-^CD16^+^ NK cells, a population with reduced effector function (60–64).

### The heterogenous CD56^dim^ *GZMK* ^-^ population includes the adaptive-like and terminally differentiated NK cells

**Figure 2:**
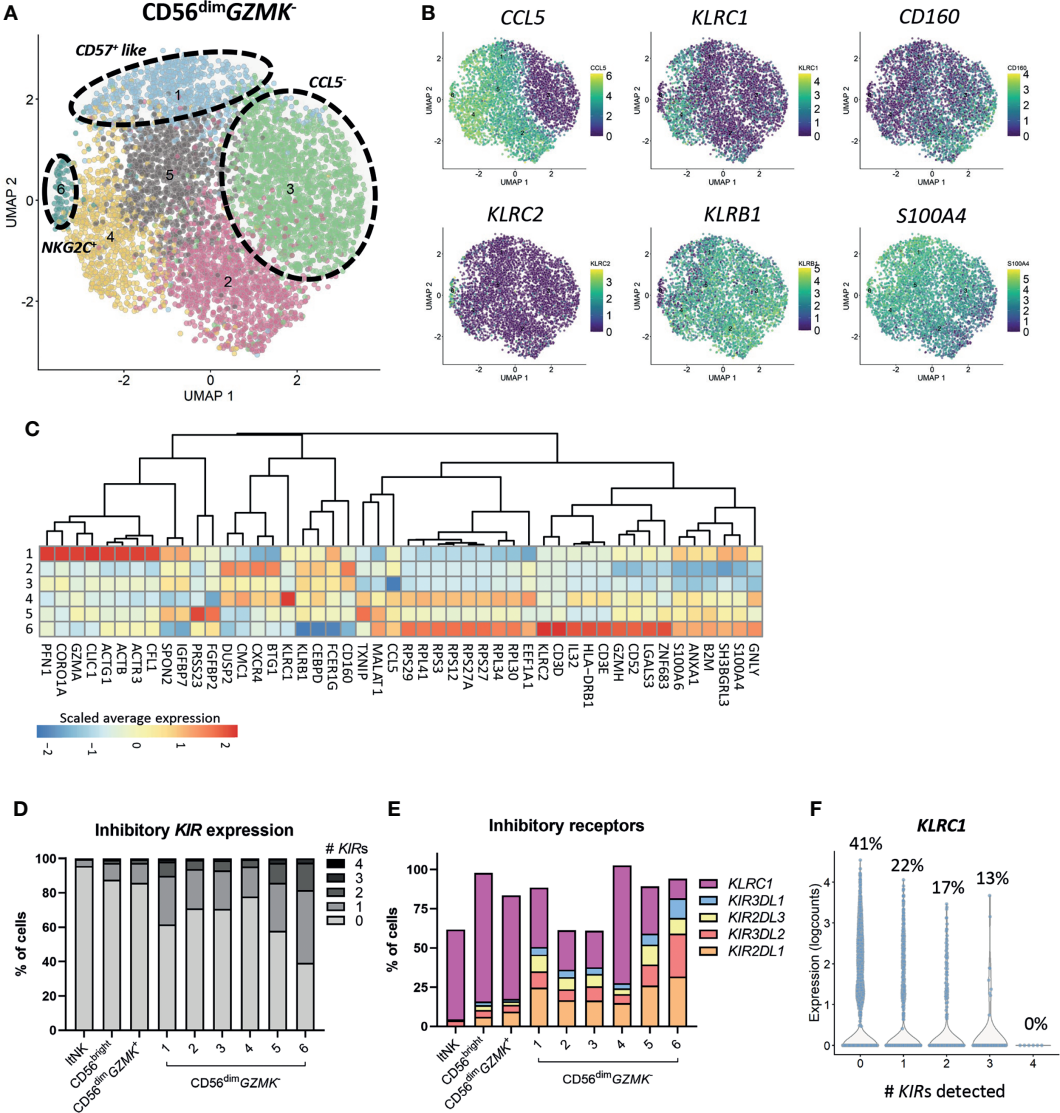
Heterogeneity of CD56^dim^ *GZMK* ^-^ population. **(A)** The CD56^dim^ *GZMK* ^-^ NK cells were selected and subclustering was performed. In total 6 clusters were identified. **(B)** The expression levels of six differentially expressed genes that were, amongst others, used to define the clusters, are shown on the UMAP plot. **(C)** For differential gene expression, each cluster was compared with each of the other clusters, using a Wilcoxon test. The top 10 most differentially expressed genes for each cluster comparison (either up- or downregulated) were selected based on statistical significance. The scaled average expression for each cluster is shown. **(D)** The percentage of inhibitory killer-cell immunoglobulin-like (*KIR)* expressing cells for each cluster was based on *KIR2DL1*, *KIR3DL2*, *KIR2DL3* and *KIR3DL1*. The detection limit for KIR gene expression was set at 0, based on the log2 normalized counts. ltNK = lymphoid tissue NK. **(E)** The expression of inhibitory receptors as percentage of total cells, per cluster is shown. **(F)** To study the relation between *KLRC1* (NKG2A) and the inhibitory *KIR*s in CD56^dim^ *GZMK* ^-^ NK cells, the expression of *KLRC1* is shown as counts and as percentage of *KLRC1* ^+^ cells, sorted by number of *KIR*s detected.

Subclustering of the CD56^bright^, ltNK and CD56^dim^ *GZMK* ^+^ cells did not reveal additional clusters, although minor heterogeneity was observed for some markers, especially *CCL5* (**Figures S2A–C**). Importantly, phenotypical markers that are often applied for further classification of the CD56^bright^ (i.e. CD117, CD27, CD16) and ltNK cells (i.e. CD16, DNAM-1, NKG2A) did not reflect distinct subsets at the transcriptional level (**Figures S2A, C**). In contrast, a high heterogeneity was observed within the CD56^dim^ *GZMK* ^-^ population, as demonstrated by seven subclusters (**Figure 2A**). Upregulated genes in cluster 2 included *DUSP2*, *CXCR4* and *BTG1*. Cluster 3 was mainly defined by absence of *CCL5* (**Figures 2A–C**). Cluster 4 separated based on the high expression of *KLRC1* (NKG2A). Cluster 5 was characterized by higher expression of *FGFBP2* and *PRSS23* (serine protease 23*)*. *S100A4* and *S100A6* were expressed at the highest level in cluster 1 and cluster 6, suggesting terminally differentiated NK cells (65–67). Unfortunately, the enzyme encoded by *B3GAT1*, creating the CD57 epitope, was not sufficiently detected. Nevertheless, genes involved in cytoskeleton remodeling (*ACTB*, *ACTG1*, *CORO1A, PFN1*) were upregulated in cluster 1, a pathway which was earlier shown to be enriched in CD56^dim^CD57^+^ NK cells to enable high cytotoxicity (**Figure 2C**) (67). The upregulation of *KLRC2* (NKG2C) in cluster 6 points to the presence of adaptive-like CMV-associated cells (68, 69). This cluster was further characterized by upregulation of ribosomal protein-coding genes, *IL32*, *GZMH* and *GNLY*, and downregulation of *KLRC1*, *KLRB1* (CD161) and *CD160* (**Figures 2B, C**).

### Inhibitory KIRs are not major drivers of CD56^dim^ *GZMK* ^-^ subclustering, but are more frequently detected in terminally differentiated cells

Although terminally differentiated cells more frequently express inhibitory KIRs, the KIRs were not included among the top 5 differentially expressed genes for each cluster comparison (**Figure 2C**) (70). Within each (sub)cluster, multiple levels of inhibitory *KIR* expression were detected (**Figure 2D**). The vast majority of the CD56^bright^ and ltNK cells did not express any *KIR*, while the intermediate CD56^dim^ *GZMK* ^+^ population expressed KIRs at levels (16%) between CD56^bright^ and CD56^dim^ *GZMK* ^-^ cells (**Figures 2D, E**). On the other hand, 35% and 56% of the terminally differentiated CD57^+^-like and *KLRC2* ^+^ cells in cluster 6 and 7, respectively, expressed at least one KIR (**Figure 2D**). Of the four inhibitory KIRs detected in our complete dataset, *KIR2DL1* was most frequently observed (**Figure 2E**). The percentage of cells expressing *NKG2A* was negatively related to the number of *KIR* molecules detected per cell, confirming previous findings (**Figures 2E, F**) (70). Overall, although the HLA-I genotype was lacking, the CD56^dim^ *GZMK* ^-^ subclusters likely did not represent unique educational states, but inhibitory *KIR*s were more frequently detected in terminal differentiated cells.

### Integration with NK cells from sixteen donors validates the identity of CD56^+^ NK cell subsets

**Figure 3:**
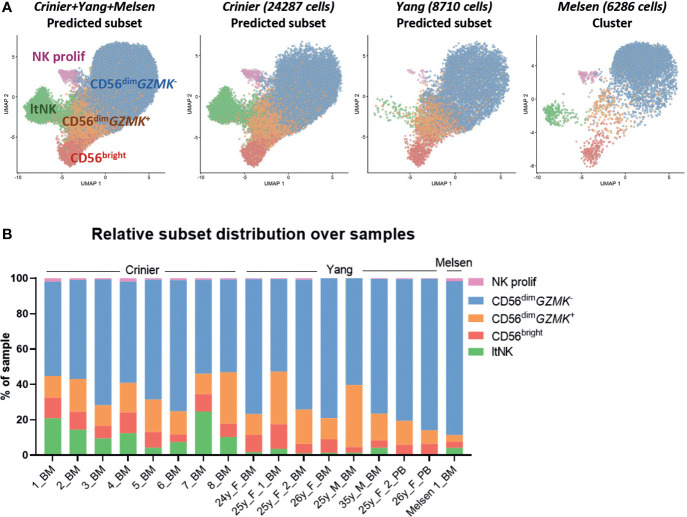
Integration of dataset with public single-cell RNA seq datasets validates clusters. **(A)** The NK cells in our dataset were selected and integrated with single-cell RNA sequencing datasets of NK cells from the Crinier dataset (GSE159624, 24287 NK cells, 8 bone marrow donors) and the Yang dataset (GSE130430, 8710 NK cells, 6 bone marrow, 2 peripheral blood donors). The UMAP was based on the corrected principal components scores. Cells were labeled using an automatic annotation method based on our own clustered dataset as reference dataset. The CD56^-^ NK cells could not be identified in the Yang and Crinier dataset, and were therefore removed from further analysis. ltNK = lymphoid tissue NK, NK prolif = proliferating NK. **(B)** The relative subset distribution within each individual sample is shown. All CD56^+^ NK cell subsets from our own dataset, were identified in the majority of the other samples as well. BM, bone marrow; PB, peripheral blood.

To validate the presence of the identified NK cell subsets in other donors, we integrated our dataset with two public single-cell RNA sequencing datasets: 24552 thawed magnetically enriched and sorted CD3^−^CD14^−^CD19^−^CD45^+^CD56^+^ NK cells (35) from bone marrow (n=8, Crinier dataset) and 9071 fresh sorted Lin^-^CD56^+/-^CD7^+^ NK cells (31) from bone marrow (n=6) and blood (n=2, Yang dataset). In these public datasets, multiple subsets of mature NK cells and low numbers of ILCs, T cells, and progenitor cells were detected (**Figure S3**). After integration with our data, we created a UMAP and annotated the cells from the public datasets using the NK cell clusters as defined in our dataset (**Figure 1A**) as reference. The UMAP of the integrated dataset containing 17 donors was highly similar to the UMAP of our individual dataset (**Figures 3A**, **1A**). Within the public datasets, the major 6 NK cell subsets could be identified based on the automatic annotation, except the CD56^-^ NK cells, indicating that this population may be donor-specific (**Figure 3A**). The CD34^+^ and ltNK cells were barely detected in blood, confirming their bone marrow residency (**Figure 3B**). Except for the proliferating NK cells, the identified CD56^+^ NK cell subsets were present in bone marrow of all individual donors (**Figure 3A**). The low number of proliferating NK cells in the Yang dataset was probably caused by an insufficient number of cells per donor included in the dataset.

Within the CD56^dim^ *GZMK* ^-^ subset of the public datasets activated NK cells (*NFKBIA*↑, *FOS*↑, *JUNB*↑) were identified in most of the donors (**Figures S3A–F**). A difference in sample handling (e.g. storage time and temperature) might have caused induction of the early response genes in the Yang and Crinier dataset, explaining the absence of activated CD56^dim^ *GZMK* ^-^ NK cells in our dataset (71). In agreement with our dataset, the terminal differentiated CD57^+^-like and *KLRC2* ^+^ NK cells were identified within the Yang dataset (**Figures S3B, D, F**). The *KLRC2* ^+^ subset contained the highest percentage of KIR-expressing cells, but again, no inhibitory KIR driven subclustering was observed (**Figures S3G, H**). Within the integrated CD56^bright^ NK cell cluster, we did not identify subclusters. Within the integrated CD56^dim^ *GZMK* ^+^ and ltNK cell population subclusters were identified based on *CCL5*, ribosomal protein genes and activation associated genes (**Figures S2D–H**). In conclusion, the identified major CD56^+^ NK cell populations in our dataset are not donor-specific confirming that they are common NK cell populations.

### CD56^dim^ *GZMK* ^+^ cells have an intermediate but distinct NKG2A^high^CD16^low^TIGIT^high^KLRG1^high^ phenotype

**Figure 4:**
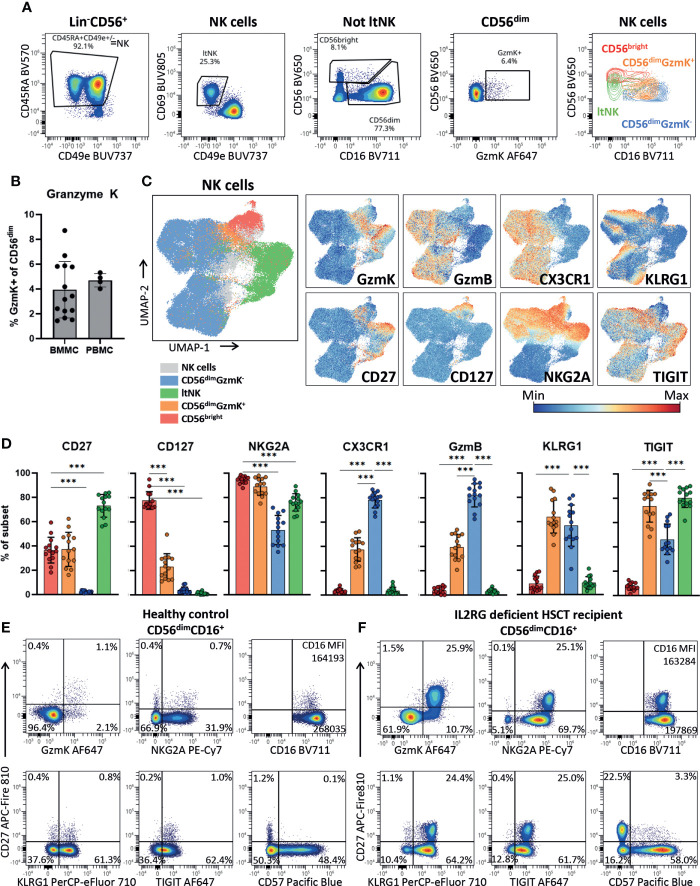
CD56^dim^GzmK^+^ cells have an intermediate but distinct phenotype. **(A)** Within the Lin^-^CD56^+^ population, NK cells were selected as CD45RA^+^CD49e^+/-^ (see **Figure S4A** for upstream gating strategy). Lymphoid tissue (lt)NK cells were defined as CD69^+^CD49e^-^. The non ltNK cells were subdivided into CD56^bright^CD16^+/-^ and CD56^dim^CD16^+^ NK cells. Next, the CD56^dim^ NK cells were subdivided into a granzyme K (GzmK)^+^ and GzmK^-^ population. The last plot is an overlay of the 4 gated NK cell subsets. One representative bone marrow donor is shown. **(B)** Quantification of the CD56^dim^GzmK^+^ cells in bone marrow mononuclear cells (BMMC, n=14) and peripheral blood mononuclear cells (PBMC, n=4). **(C)** The UMAP embedding of 91079 NK cells from 14 thawed BMMC healthy control samples, based on 21 NK cell markers (**Figure S4B**). The expression of a selection of markers is shown. GzmB = granzyme B. **(D)** Expression of a selection of markers on bone marrow NK cell subsets (n=14). Color legend is embedded in C. The mean and SD are indicated in the bar graphs. Repeated measures one-way ANOVA was applied for statistical testing with Dunnett’s correction for multiple testing. For CD27, CD127 and NKG2A, CD56^bright^ NK cells were set as reference, while for the remaining markers the CD56^dim^GzmK^-^ NK cells were set as reference. Adjusted P-value <0.001*** **(E)** Expression of a selection of markers plotted against CD27 on CD56^dim^CD16^+^ NK cells of a representative healthy blood donor and **(F)** IL2RG deficient patient that received a hematopoietic stem cell transplantation (HSCT) 51 years ago. In the patient, an enrichment of the CD56^dim^GzmK^+^ cells was observed.

To validate the presence of the newly identified CD56^dim^ *GZMK* ^+^ NK cells using protein expression and further phenotypically profile them, we developed a 26-marker panel for spectral cytometry. Indeed, we identified the CD56^bright^, CD56^dim^GzmK^+^, CD56^dim^GzmK^-^ and ltNK cells among the NK cells in bone marrow of 14 healthy donors (**Figures 4A**, **S4A**). In the classical two-dimensional plot of CD56 against CD16, the CD56^dim^GzmK^+^ cells (orange) are positioned between the CD56^bright^ and CD56^dim^GzmK^-^ population. A mean of respectively 3.9% and 4.7% of CD56^dim^ NK cells in bone marrow and blood, expressed granzyme K (**Figure 4B**). The UMAP embedding of the NK cells from 14 healthy bone marrow donors, based on 21 NK cell markers, was comparable to the UMAP of the transcriptomic data, with the CD56^dim^GzmK^+^ subset positioned between the other three NK cell subsets (**Figure 4C**; **Figure S4B**). The phenotype of CD56^dim^GzmK^+^ cells was characterized by variable expression of CD56^bright^-related markers CD127, CD27 and CD56^dim^-related markers CX3CR1 and granzyme B (**Figures 4C–E**). The vast majority of CD56^dim^GzmK^+^ cells expressed high levels of NKG2A and TIGIT, two markers also expressed by ltNK cells (**Figures 4D, E**). KLRG1, usually associated with mature CD56^dim^ NK cells, was also highly expressed by CD56^dim^GzmK^+^ cells, underscoring the distinctiveness of this intermediate NK cell subset. No difference in phenotype of CD56^dim^GzmK^+^ cells between blood and bone marrow was found (**Figure S4C**). Interestingly, in an IL2RG deficient patient who received a hematopoietic stem cell transplantation (HSCT) more than 50 years ago and is affected with chronic HPV disease, 26% of the CD56^dim^ NK cells expressed granzyme K and CD27 (**Figure 4F**) (72). While a similar CD56^dim^CD27^+^ subpopulation with a GzmK^+^NKG2A^high^CD16^low^TIGIT^high^KLRG1^high^ phenotype was also present at a low frequency in healthy controls, it was significantly expanded in this post HSCT patient (**Figures 4D–F**). Combined, the high expression of KLRG1 and TIGIT, and expansion in a specific clinical condition, suggest that CD56^dim^GzmK^+^ NK cells represent an intermediate but discrete stage during NK cell differentiation.

### CD56^dim^GzmK^+^ cells produce intermediate levels of chemokines and cytokines upon interleukin or target cell stimulation

**Figure 5:**
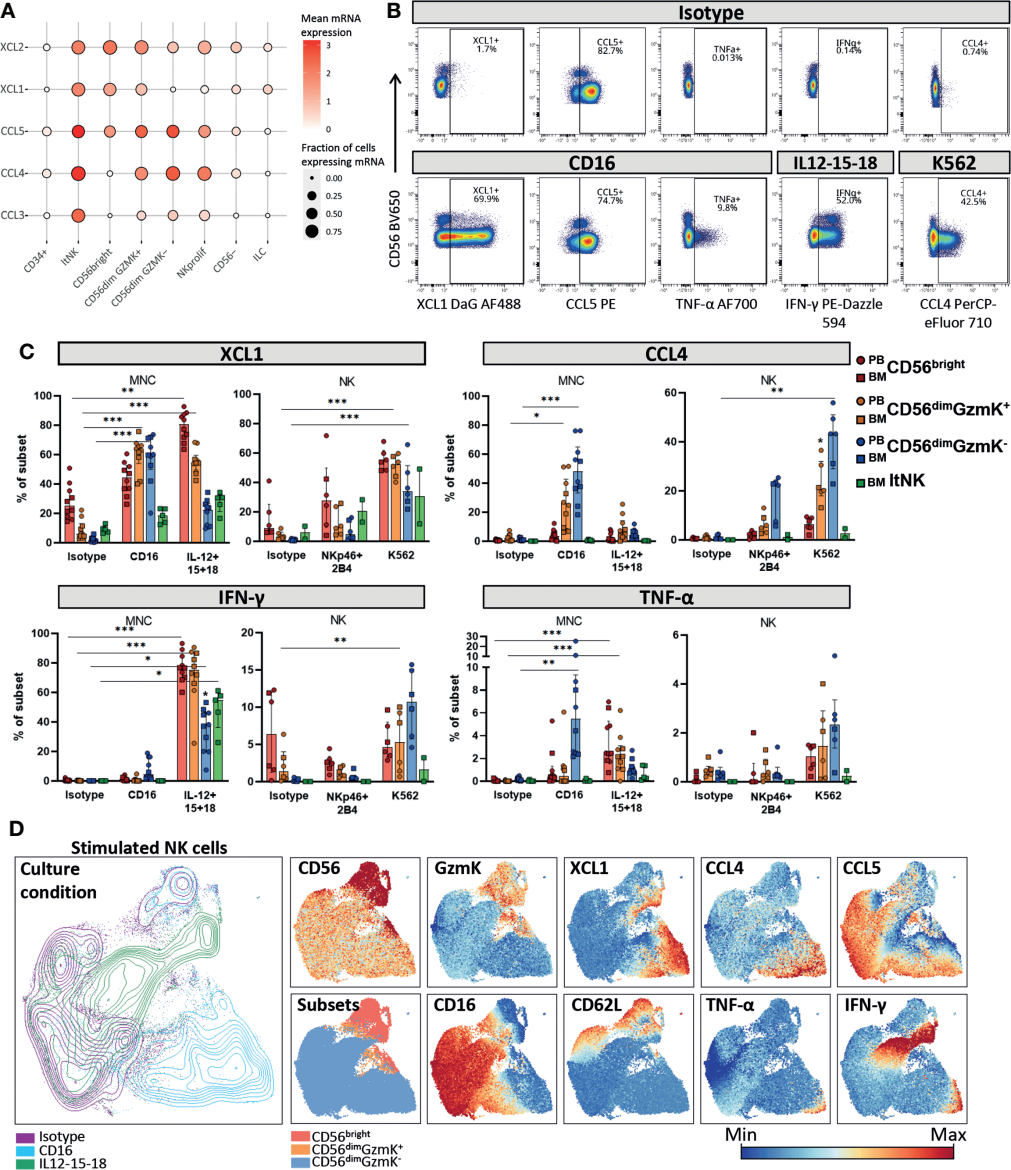
Chemokine and cytokine production by NK cell subsets. **(A)** Dotplot visualization of chemokine mRNA expression per cell subset. Shown is the mean mRNA expression (color intensity) and the fraction of cells of each subset expressing the mRNA (size). ltNK = lymphoid tissue NK, NK prolif = proliferating NK, ILC = innate lymphoid cells. **(B)** Intracellular expression of chemokines and cytokines in NK cells cultured in the presence or absence of stimulation, as measured by spectral cytometry. A representative blood donor is shown. **(C)** NK cells were cultured as bulk thawed mononuclear cells (MNC, n=5 blood, n=5 bone marrow) or as freshly enriched NK cells (n=4 blood, n=2 bone marrow). MNC were stimulated for 4 hours and enriched NK cells were stimulated overnight, by cytokines or target-cell(-like) stimulation. The intracellular effector molecule production was determined in the gated peripheral blood (PB) and bone marrow (BM) derived NK cell subsets. Bars indicate median and interquartile range. A paired Friedman test was applied to test for differences with isotype control for each subset. Adjusted P-value, *<0.05, **<0.01, ***<0.001. **(D)** NK cells from PBMC stimulated for four hours with IL12-15-18 (green), anti-CD16 (blue), or isotype control (purple) were embedded in a UMAP. The protein expression of several markers is indicated on the UMAP plot. A representative donor is shown.

The identification of the CD56^dim^GzmK^+^ NK cell subset raised the question on its functional capacity compared to the CD56^bright^, CD56^dim^GzmK^-^ and ltNK subset. Based on the transcriptome, the CD56^dim^ *GZMK* ^+^ cells had an intermediate chemokine profile (based on *CCL3*, *CCL4*, *CCL5*, *XCL1* and *XCL2)* matching characteristics of both CD56^bright^, CD56^dim^ *GZMK* ^-^ cells and ltNK cells (**Figure 5A**). ltNK cells had the highest chemokine expression, with each chemokine detected in at least 85% of the cells, while CD56^bright^ NK cells had the lowest overall chemokine expression, with absence of *CCL3* and *CCL4* expression (**Figure 5A**). Unfortunately, *IFNG* and *TNF* were underrepresented in our dataset. Notably, all those effector molecules, including *IFNG* and *TNF*, were previously shown to be expressed in each major NK cell subset, by bulk mRNA sequencing (**Figure S5A**) (20).

The mRNA levels for cytokines were determined under steady state conditions. To study whether XCL1, CCL4, CCL5, IFN-γ and TNF-α were also produced at the protein level by each individual NK cell subset we stimulated NK cells in bulk mononuclear cells (MNC), or as an enriched NK fraction. In order to include an appropriate stimulation for each subset, we stimulated the cells with either interleukins (IL-12, IL-15, IL-18) or target-cell(-like) stimulations (anti-CD16, anti-NKp46 & anti-2B4 or K562 cells, **Figures S5B**, **5B**). XCL1 was abundantly produced by all subsets in response to all stimuli (**Figure 5C**). In contrast, CCL4 and TNF-α were mainly produced by CD56^dim^GzmK^-^ cells in response to target-cell(-like) stimulations, with a median of 48% and 5.5% positivity upon CD16 crosslinking, respectively. The highest IFN-γ production was observed upon interleukin stimulation in all subsets (**Figure 5C**). Distinct from the other effector molecules, CCL5 was spontaneously produced by a fraction of each NK cell subset upon culture, likely reflecting the *CCL5* based subclustering and UMAP embedding of the transcriptomic data (**Figures 5B**; **S5C**). In line with literature, CD56^dim^GzmK^-^ NK cells were overall most responsive to target-cell(-like) stimulations, while CD56^bright^ NK and ltNK cells were most responsive to interleukin stimulation (1, 4). CD56^dim^GzmK^+^ NK cells produced effector molecules at levels in between the production by CD56^bright^ and CD56^dim^GzmK^-^ NK cells in response to both interleukins and target cell stimulation, reinforcing their position as an intermediate subset (**Figure 5C**).

To study the repertoire of produced effector molecules at the single-cell level we embedded NK cells cultured for four hours with either isotype or anti-CD16 antibodies, or a combination of IL-12, IL-15 and IL-18 in a UMAP. Within the anti-CD16 responding CD56^dim^GzmK^-^ NK cells (blue, lower right corner), we observed a higher frequency of cells positive for XCL1 and CCL4, compared to IFN-y and TNF-α. This can be explained by the fact that chemokines are produced earlier compared to cytokines (1) (**Figure 5D**). Nevertheless, the cells that produced IFN-γ and TNF-α, also produced XCL1 and CCL4, indicating that the repertoire of effector molecules is similar among stimulated NK cells. The same phenomenon was observed for the CD56^bright^ NK cells in response to interleukins: the IFN-γ producing cells also produced XCL1 (green, upper right corner, **Figure 5D**).

### A shift from granzyme K to granzyme B characterizes circulating NK cell differentiation

**Figure 6:**
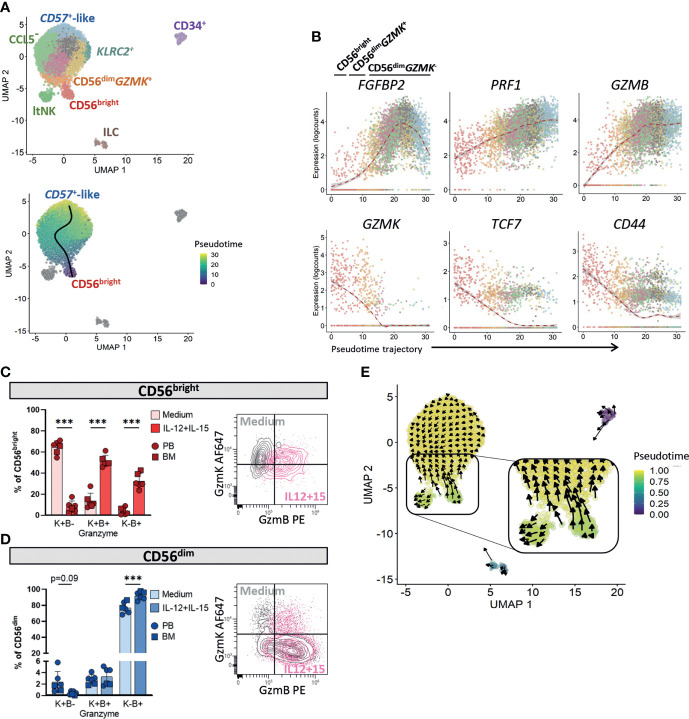
Pseudotime analysis in bone marrow suggests that ltNK cells develop independently from circulating NK cells. **(A)** Pseudotime analysis was performed on our NK cell dataset using Slingshot, based on the 50 principal components and 6 clusters (CD56^bright^, CD56^dim^ *GZMK* ^+^, CD56^dim^ *GZMK* ^-^, lymphoid tissue (lt)NK, CD34^+^ and innate lymphoid cells (ILCs)). In the upper panel, the subclusters are visualized on the UMAP plot. Only one trajectory was detected, connecting the CD56^bright^, CD56^dim^ *GZMK* ^+^ and CD56^dim^ *GZMK* ^-^ NK cells (lower panel). The other clusters did not form a trajectory. **(B)** Six genes of which the expression changed differentially among pseudotime of the CD56^bright^-CD56^dim^ trajectory are shown. Colors represent subclusters, as in A. **(C, D)** Fresh mononuclear cells from peripheral blood (PB, n=4) and thawed mononuclear cells from bone marrow (BM, n=2) were either rested or stimulated overnight with IL-12 and IL-15 to study intracellular granzyme K (GzmK) and granzyme B (GzmB) production. For **(C)** the CD56^bright^ NK cells and **(D)** the CD56^dim^ NK cells, the GzmK and GzmB expressing cells were subdivided into three populations: (granzyme)K+B-, K+B+, and K-B+. The density plot of a representative blood donor is shown. The CD56^bright^ and CD56^dim^ NK cell populations were gated as shown in **Figure 4A**. A paired one-way ANOVA was performed with Šidák correction for multiple testing. ***P-value <0.001. The bargraphs represent mean and SD. **(E)** RNA velocity analysis was performed as alternative method for pseudotime analysis. The length of the arrow represents the velocity (transcription rate) and the direction of the arrow points to the predicted future state.

To study the developmental relationship between the mature NK cell subsets CD56^bright^, CD56^dim^GzmK^+^, CD56^dim^GzmK^-^ and ltNK cells in bone marrow, we performed pseudotime analysis on our transcriptomic dataset using the Slingshot algorithm. The proliferating NK cells were excluded since this cluster represented a mixed population of CD56^bright^ and CD56^dim^ NK cells (**Figure 1B**). The CD56^-^ NK cells were not identified in other donors, and therefore removed as well. Slingshot analysis identified only one trajectory connecting the CD56^bright^, CD56^dim^ *GZMK* ^+^ and CD56^dim^ *GZMK* ^-^ NK cells (**Figure 6A**). The CD56^bright^ NK cells were positioned at one end, while the terminally differentiated CD57^+^-like NK cells were positioned at the other end of the principle curve. Therefore, we considered those as most differentiated cells, the CD56^bright^ NK cells as starting subset and the CD56^dim^ *GZMK* ^+^ NK cells as intermediate subset. Among the most differentially expressed genes driving the pseudotime of the circulating NK cell trajectory, we identified effector molecules *FGFBP2*, *PRF1*, *GZMB*, *GZMK*, the transcription factor *TCF7*, and the adhesion molecule *CD44* (**Figure 6B**). To further study the shift from granzyme K towards granzyme B, we cultured PBMC and BMMC with IL-12 and IL-15. For both the CD56^bright^ and CD56^dim^ NK cells, we observed a decrease in GzmK^+^GzmB^-^ cells, and increase in GzmK^-^GzmB^+^ cells after stimulation, supporting a shift from granzyme K to B during NK cell differentiation (**Figures 6C, D**).

### Pseudotime analysis suggests that ltNK develop independently from circulating NK cells

The slingshot analysis indicated that ltNK cells are not developmentally connected to the circulating NK cells. To validate this by another pseudotime algorithm we applied RNA velocity analysis. Whereas arrows of the CD56^bright^ NK cells pointed towards the CD56^dim^ NK cells in their predicted future state, the arrows of the ltNK cells did not point towards CD56^bright^ nor CD56^dim^ NK cells (**Figure 6E**). Moreover, the pseudotimes of ltNK and CD56^bright^ NK cells were comparable (**Figure 6E**). By decreasing the minimum distance parameter of the UMAP, the CD56^bright^ NK cells were still connected to the CD56^dim^ *GZMK^-^* NK cells *via* the CD56^dim^ *GZMK* ^+^ subset, while the ltNK cells were not (**Figure S6**). Together, these results suggest that ltNK cells develop independently from the circulating NK cells and do not differentiate into a circulating NK cell subset. Still, there might be NK cell progenitors that give rise to both the ltNK cells and the other NK cell subsets.

### No evidence for NK cell development in human bone marrow at steady state

**Figure 7:**
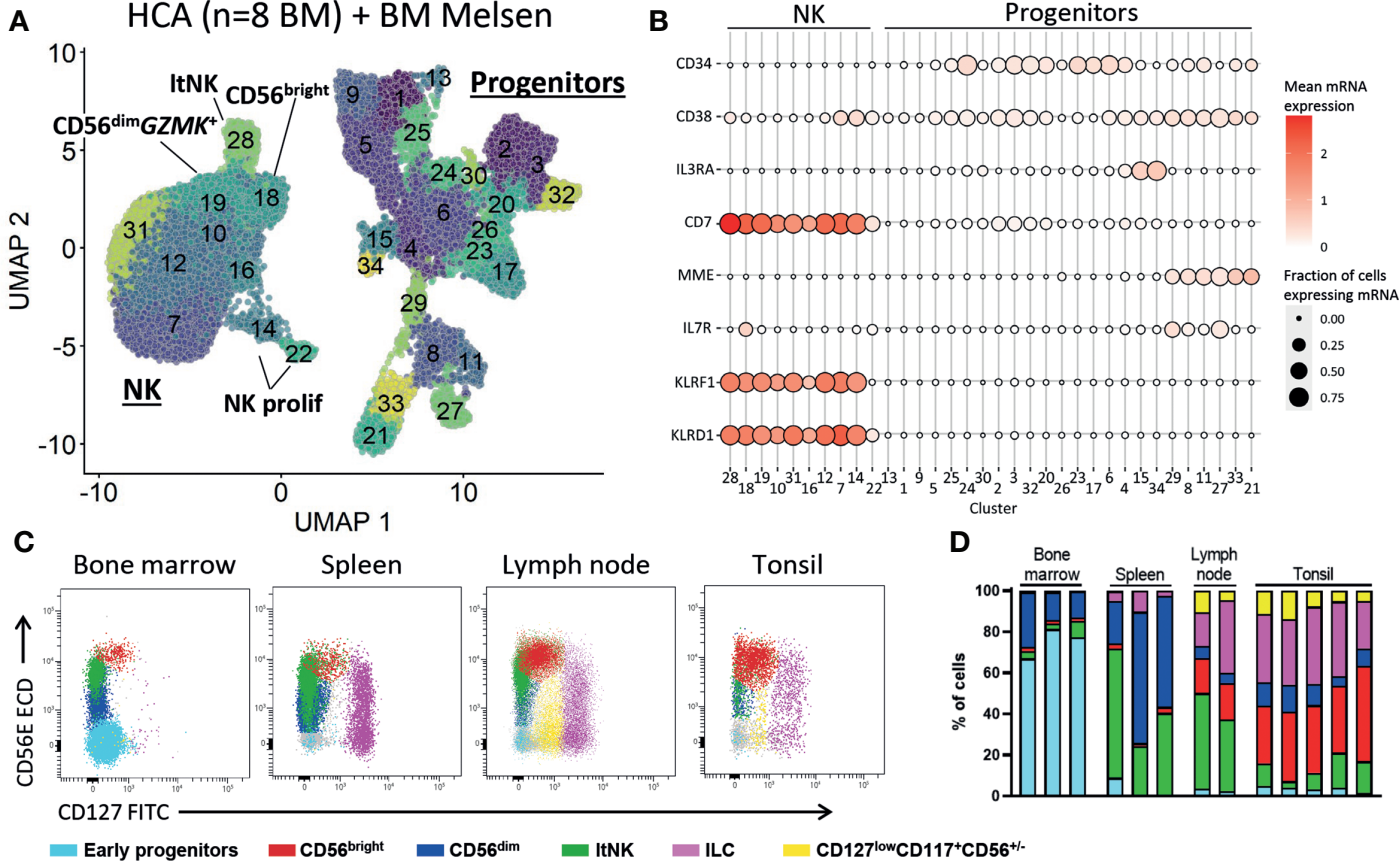
No early NK cell stages detected in bone marrow. **(A)** To study early NK cell development, our dataset was integrated with the human cell atlas (HCA) bone marrow (BM) single-cell RNA sequencing dataset (8 donors). Progenitor and NK cells were selected and re-clustered (total = 41476 cells). See **Figure S8** for the selection procedure. ltNK = lymphoid tissue NK, NK prolif = proliferating NK. **(B)** For each cluster as visualized in **A**, the mean mRNA expression (color intensity) and the fraction of cells expressing the mRNA molecule (size) are shown. A selection of progenitor-related genes is depicted. No NK progenitor cluster with combined expression of *CD34*, *CD38*, *CD7* and *MME* (CD10) could be identified. **(C)** Fresh mononuclear cells isolated from bone marrow (n=3), lymph node (n=2), tonsil (n=5) and spleen (n=3) were analyzed for the presence of NK cell development. First, T cells, B cells and monocytes were excluded (**Figure S9**). Next, the early progenitor cells (CD45^-/dim^CD117^+^SSC^high^) and NK/ILC/progenitor cells were defined based on a UMAP embedding (**Figure S9**). Within the NK/ILC/progenitor cells, CD56^bright^ NK, CD56^dim^ NK, ltNK cells, and innate lymphoid cells (ILCs, CD127^high^CD117^+^) were recognized. In the remaining cells, a population of CD127^low^CD117^+^CD56^+/-^ cells was identified (yellow), present in lymph node and tonsil, but absent in spleen and bone marrow. **(D)** subset distribution in the individual tissue samples.

The presence of CD34^+^ cells in our dataset allowed us to explore early NK cell development. We re-clustered the CD34^+^ cells and identified multiple lineage committed and progenitor cells guided by the cell subset scores based on reference bulk RNA sequence data (52) (**Figures S7A, B**). Since there were too few NK progenitor cells to reliably model the NK cell development we integrated our dataset with 316941 bone marrow cells from the human cell atlas. All hematopoietic populations were identified, including progenitor cells (cluster 6, 18, 24, 28, 29) and NK cells (cluster 3, 30, **Figures S8A, B**). The myeloid, erythroid and B cell development was evident by the presence of intermediate cell stages connecting the progenitor cells and respective mature populations (**Figure S8A**). However, the T and NK cells were not connected to the progenitor cells (**Figure S8A**). This suggests that, like T cells, NK cells develop outside the bone marrow niche. To further study this, we selected the progenitor and NK cells and re-clustered the data (**Figure 7A**). Also, in the new UMAP embedding of these cells we found no connection between the NK and the progenitor cell clusters, suggesting that there were no cells with characteristics representative for early NK cell stages (**Figure 7A**; **Figure S8C**). The common lymphoid progenitors (CLP) were identified in cluster 8, 11, 27 and 29 by automatic annotation (**Figure S8C**) and manual annotation (*CD34* ^+^ *CD10* ^+^ *IL7R* ^+^ *CD38* ^+^, **Figure 7B**). However, we found no cluster with combined expression of *CD34*, *CD38*, *CD7* and *MME* (CD10), which has been previously proposed as human NK progenitor cell (**Figure 7B**) (73). Multiple articles postulated that development of NK cells occurs in secondary lymphoid organs (74–76). In agreement, we demonstrate that lymph node and tonsil, but not bone marrow and spleen, harbor a cell population with an early NK cell phenotype (CD127^low^CD117^+^CD56^+/-^) that is absent from bone marrow and spleen (**Figures 7C, D**, **Figure S9**). Moreover, the less mature CD56^bright^ NK cell subset was highly enriched in tonsil and lymph node, compared to bone marrow and spleen (**Figure 7D**). In conclusion, these findings provide evidence that NK cell development occurs in tonsil and lymph node, rather than in bone marrow.

## Discussion

Single-cell RNA sequencing combined with antibodies of human bone marrow NK cells revealed a CD56^dim^ NK cell subpopulation characterized by expression of granzyme K. The transcriptional, phenotypical and functional profiles of the minor CD56^dim^GzmK^+^ population were intermediate between the CD56^bright^ and CD56^dim^GzmK^-^ subset. Pseudotime analysis positioned CD56^bright^, CD56^dim^GzmK^+^ and CD56^dim^GzmK^-^ cells in one differentiation trajectory, while ltNK cells were developmentally separated. Progenitor NK cells could not be identified in bone marrow, suggesting that other lymphoid tissues are responsible for NK cell development.

Using trajectory analysis on single-cell RNA sequence data, we confirmed that the CD56^bright^ NK cells are precursors of the CD56^dim^ NK cells (31, 34, 35). Clustering and trajectory analysis of our single-cell RNA sequence data, and phenotypical and functional analysis identified CD56^dim^GzmK^+^ cells as intermediate subset, with a CD27^+/-^GzmB^low^CX3CR1^low^NKG2A^high^CD16^low^KLRG1^high^TIGIT^high^ phenotype. In literature, multiple populations have been proposed as intermediate differentiation stage, based on flow cytometry data: CD56^bright^CD16^+^, CD56^bright^CD27^-^, CD56^dim^CD94^high^, CD56^dim^CD62L^+^ and CD56^dim^CD16^dim^ (24, 26–29, 77). Phenotypically, the CD56^dim^GzmK^+^ subset resembles the proposed CD56^dim^CD94^high^ and CD56^dim^CD16^dim^ NK cell subsets (28, 77, 78). However, in contrast to the gradual differences in CD94 and CD16 expression, the bi-modal expression of granzyme K within the CD56^dim^ NK cells allows for strict gating of populations. Moreover, in parallel to CD8 T cells, conversion of granzyme K to granzyme B was driving the differentiation in pseudotime analysis (79–81). Therefore, we consider CD56^dim^GzmK^+^ cells as an intermediate NK cell subset. The question arises whether this subset represents a continuum of cells differentiating towards the CD56^dim^GzmB^+^ subset, or whether it represents a defined differentiation stage. The fact that this subset was expanded in an IL2RG deficient patient post HSCT, but also uniformly expressed high levels of both KLRG1 and TIGIT, is in favor of a defined differentiation stage.

By subclustering of the CD56^dim^GzmK^-^ NK cells, the known terminally differentiated CD57^+^ and adaptive-like NKG2C^+^ NK cells were most notably different. The functional response of CD56^dim^ NK cells is influenced by the educational state, i.e. the expression of self-inhibitory KIRs and NKG2A (70, 82). Moreover, CD56^dim^ NK cell differentiation is associated with loss of NKG2A and sequential gain of KIR expression (70). Indeed, in our spectral cytometry data, NKG2A and KIR expression were driving the UMAP embedding, however this was not obvious from the transcriptome-based pseudotime analysis or clustering, except for the lowest and highest inhibitory *KIR* expression observed in a minor *KLRC1* ^high^ cluster and *KLRC2* ^+^ cluster, respectively. As previously reported, no difference in NKG2A^+^ and NKG2A^-^ CD56^dim^ NK cells was observed by bulk RNA sequencing, and increase of cytotoxicity in educated NK cells was shown to be independent of transcription (20, 83). Thus, although the single-cell RNA sequencing results suggest that expression of genes encoding for the inhibitory receptors are not the main drivers of CD56^dim^ NK cell subset classification, the additional use of KIR-specific oligonucleotide-conjugated antibodies in future experiments will be essential in understanding the NK cell education in relation to the process of differentiation.

Although all the NK cell subsets in this study were identified by transcriptome-based clustering, the use of oligonucleotide-antibodies specific for CD56 and CXCR6 was of added value to recognize the tissue-resident ltNK cells in our data set. Previously, this population was unrecognized by single-RNA sequencing on bone marrow NK cells (31, 35, 84). Like the CD56^bright^ NK cells, the ltNK cells express granzyme K, but not granzyme B, perhaps explaining their low cytotoxicity compared to CD56^dim^ NK cells at resting state (4, 21). The role of granzyme K in NK cells is still poorly understood. Both intracellular and extracellular roles have been suggested for granzyme K, including inhibition of viral replication and induction of pro-inflammatory cytokines, respectively (85). The highest functional response of ltNK and CD56^bright^ NK cells was observed upon interleukin stimulation, as shown by XCL1 and IFN-y production. Although ltNK cells expressed the highest mRNA levels of multiple effector molecules (20), including CCL4 and TNF-a, neither our target-cell or antibody stimulation, nor interleukin stimuli induced the highest production in ltNK cells compared to the other subsets. It requires further investigation to decipher the appropriate physiological stimuli and optimal duration of *in vitro* stimulation for ltNK cells.

In agreement with murine data on tissue-resident NK cells, the ltNK cells likely develop independently from the circulating NK cells based on trajectory analysis (86). Although we hypothesized that the ltNK and CD56^bright^ NK cells diverge from a shared NK cell progenitor, we were not able to decipher the *in situ* development of ltNK cells nor the CD56^bright^ NK cells, since no NK precursor cells were identified in bone marrow from healthy donors. The hypothesis that bone marrow is the site for human NK cell development originates mainly from *in vitro* studies, where early bone marrow-derived progenitor cells were sorted and cultured in the presence of growth factors, interleukins and/or bone marrow stromal cells to generate cytotoxic NK cells (87, 88). The current model describes that hematopoietic stem cells give rise to common lymphoid progenitor cells (CLP), which subsequently downregulate CD34 and upregulate CD56 (89). However, the exact sequential steps especially in early NK cell development are not completely understood. An NK-lineage restricted progenitor has been described in human bone marrow (73), but we were neither able to identify these cells, nor did we identify intermediate stages linking CD34^+^ progenitors to mature NK cells in human bone marrow. In contrast, in the lymph node and tonsil, after exclusion of ILCs, CD127^low^CD117^+^CD56^+/-^cells were identified that might represent the direct precursors of the CD56^bright^ NK and ltNK cells. Multiple reports provided evidence for the presence of NK progenitor cells and intermediate stages in secondary lymphoid tissues (74–76, 90, 91), but the appropriate markers to define these cells remain yet to be identified. Therefore, it would be interesting to decipher the NK cell development by single-cell trajectory analysis of RNA and protein data in these tissues.

Overall, the use of untouched fresh NK cells (including CD34^+^ cells) and oligonucleotide-labeled antibodies, resulted in a unique single-cell RNA sequencing dataset of high quality based on the number of genes and UMIs detected per cell. Still, the detection limit of single-cell RNA sequencing compromises studying markers with limited mRNA expression, highlighting the additional value of using oligonucleotide-conjugated antibodies, and validation by other techniques. Although we performed single-cell RNA sequencing on one healthy donor, data integration with public datasets including 16 donors, and spectral cytometry on 14 donors validated our results.

In conclusion, we provide detailed analyses on single-cell RNA sequence data of human bone marrow NK cells. Our work challenges the current statement that NK cell development occurs in bone marrow, proposes that tissue-resident ltNK cells develop independently from circulating NK cells, and define CD56^dim^GzmK^+^ NK cell population as an intermediate stage in NK cell differentiation.

## Data availability statement

The data presented in the study are deposited in the GEO repository, accession number GSE199411. The data can be found here: https://www.ncbi.nlm.nih.gov/geo/query/acc.cgi?acc=GSE199411.

## Ethics statement

The studies involving human participants were reviewed and approved by the Institutional Review Board (protocols P00.068, P01.028, B17.001 and LUMC healthy voluntary donor service (LuVDS)). The patients/participants provided their written informed consent to participate in this study. Written informed consent was obtained from the individual(s) for the publication of any potentially identifiable images or data included in this article.

## Author contributions

JM designed the study, performed experiments, analysed the data and wrote the manuscript. MD and DH designed the study, conducted experiments and analysed the data. DS, PS and DH performed experiments and analysed the data. GL performed experiments and analysed data. AL, GL and MS supervised the study and wrote the manuscript. All authors contributed to the article and approved the submitted version.

## Funding

JM was supported by funding from the Leiden University Medical Center, the graduate program of Nederlandse Organisatie voor Wetenschappelijk Onderzoek and Stichting Zeldzame Ziekten Fonds (SCID project).

## Acknowledgments

The authors thank Dr. Susan Kloet, Yavuz Ariyurek and Emile de Meijer from the Leiden Genome Technology Center (Leiden University Medical Center, LUMC) for technical assistance of the library preparation. The authors acknowledge Flow cytometry Core Facility of LUMC, coordinated by Dr. Koen Schepers and Marjolijn Hameetman, for facilitating the use of flow cytometers (https://www.lumc.nl/research/facilities/fcf). The authors thank Dr. Pauline van Schouwenburg (LUMC) and Martijn Cordes (LUMC) for the critical discussion on the single-cell RNA sequencing data analysis.

## Conflict of interest

The authors declare that the research was conducted in the absence of any commercial or financial relationships that could be construed as a potential conflict of interest.

## Publisher’s note

All claims expressed in this article are solely those of the authors and do not necessarily represent those of their affiliated organizations, or those of the publisher, the editors and the reviewers. Any product that may be evaluated in this article, or claim that may be made by its manufacturer, is not guaranteed or endorsed by the publisher.
