# Supplementary material for: Single-cell transcriptomics in bone marrow delineates CD56dimGranzymeK+ subset as intermediate stage in NK cell differentiation
Source: Front Immunol. 2022 Nov 24;13:1044398. doi: 10.3389/fimmu.2022.1044398 (PMC9730327; doi:10.3389/fimmu.2022.1044398)
Supplement: Supplementary file 1 [file DataSheet_1.pdf]

Supplementary Material

1 Supplementary Figures and Tables

1.1 Supplementary Figures

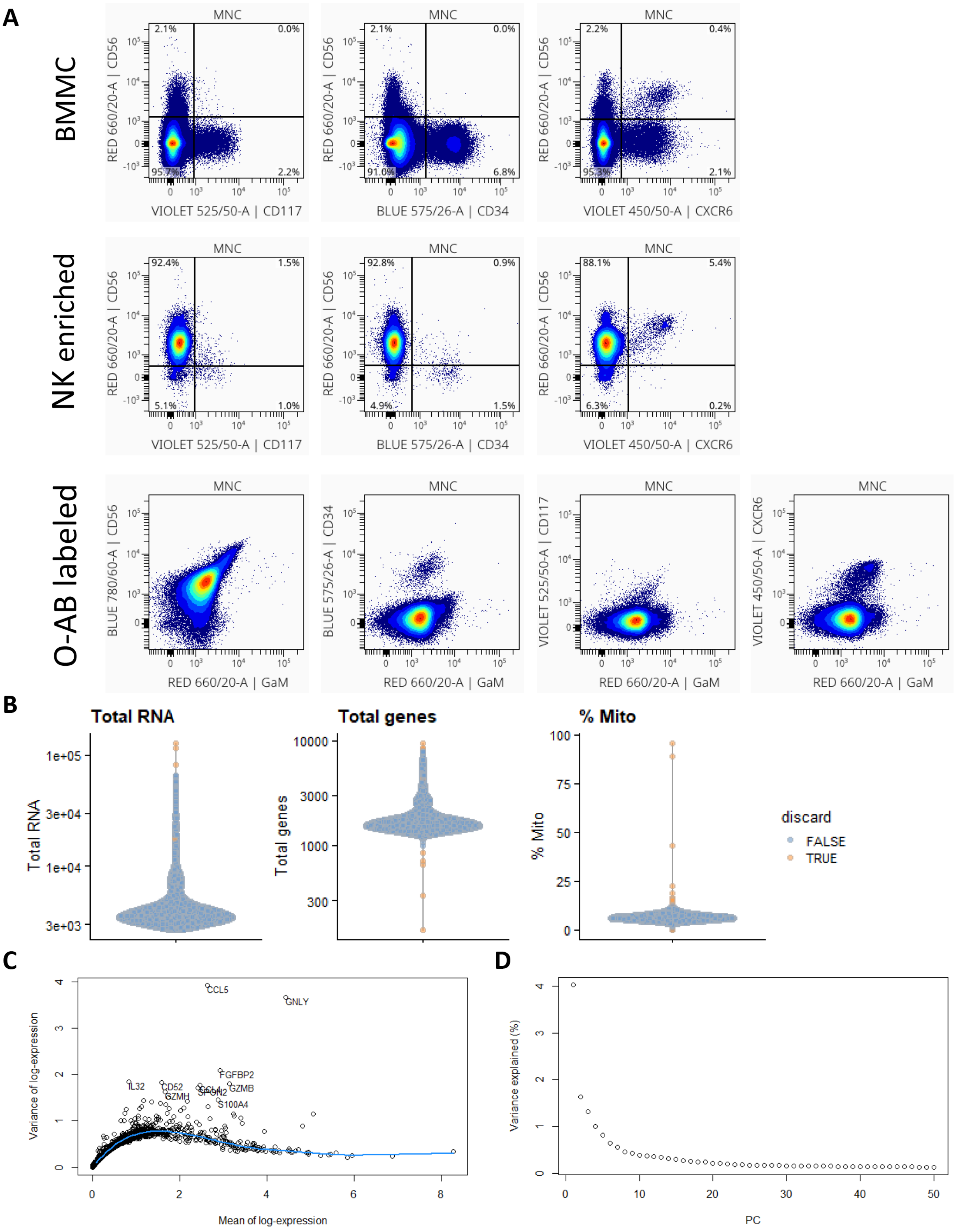
A
BMMC
NK enriched
O-AB labeled
MNC
RED 660/20-A | CD56
VIOLET 525/50-A | CD117
BLUE 575/26-A | CD34
VIOLET 450/50-A | CXCR6
BLUE 780/60-A | CD56
BLUE 575/26-A | CD34
VIOLET 525/50-A | CD117
VIOLET 450/50-A | CXCR6
RED 660/20-A | GaM
2.1%
0.0%
95.7%
2.2%
2.1%
0.0%
91.0%
6.8%
2.2%
0.4%
95.3%
2.1%
92.4%
1.5%
5.1%
1.0%
92.8%
0.9%
4.9%
1.5%
88.1%
5.4%
6.3%
0.2%
B
Total RNA
Total genes
% Mito
discard
FALSE
TRUE
C
Variance of log-expression
Mean of log-expression
CCL5
GNLY
FGFBP2
IL32
CD52
GZMH
SPON2
GZMB
S100A4
D
Variance explained (%)
PC

Figure S1. Quality control and preprocessing single-cell RNA sequencing

(**A**) Bone marrow mononuclear cells (BMMC) were negatively enriched for NK cells and labeled with oligonucleotide-conjugated antibodies (O-AB) against CD56, CXCR6, CD117 and CD34. By flow cytometry the composition of each fraction was tested. The O-AB labeled NK cells were stained with APC-conjugated goat-anti-mouse (GaM), followed by fluorochrome-conjugated antibodies against CD56, CD34, CD117 and CXCR6 to validate the binding of the oligonucleotide-conjugated antibodies. **(B)** Low-quality cells with <1000 expressed genes, >8300 expressed genes and >12.5% mitochondrial RNA were removed from further analyses (in total 21 cells). 454 remaining doublets were removed that clustered based on high mRNA and gene content. In total, 6525 cells were included in subsequent analyses. **(C)** After log-transformation and normalization, the top 2000 most variable genes were selected by computing the variance of the log-counts and fitting a trend to the variance with respect to abundance across the genes. The top 10 most variable genes are marked. **(D)** Principal component (PC) analysis was applied based on the top 2000 most variable genes. The top 20 PCs, explaining 14.2% of the variance, were retained for further analyses.

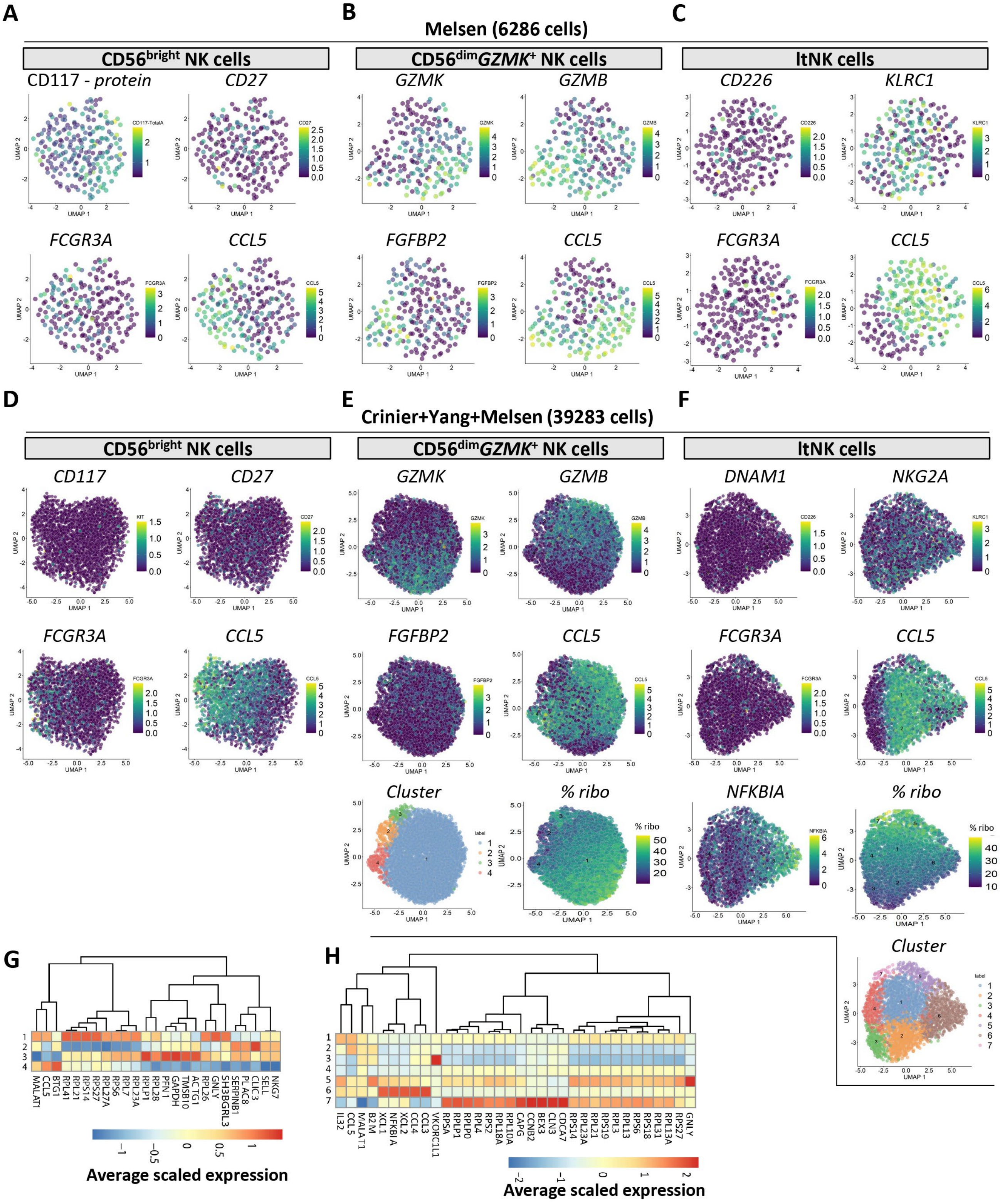
A
B
Melsen (6286 cells)
C
CD56bright NK cells
CD56dimGZMK+ NK cells
ltNK cells
CD117 - protein
CD27
GZMK
GZMB
CD226
KLRC1
FCGR3A
CCL5
FGFBP2
D
E
Crinier+Yang+Melsen (39283 cells)
F
CD117
DNAM1
NKG2A
Cluster
% ribo
NFKBIA
G
H
Average scaled expression

Figure S2. Subclustering of CD56^{bright}, CD56^{dim}*GZMK*$^{+}$ and ltNK cells

(A) CD56^{bright} NK cells (n=228), **(B)** CD56^{dim}*GZMK*$^{+}$ NK cells (n=225) and **(C)** lymphoid tissue (lt)NK cells (n=258) were selected and further subclustered (k=8) based on the first 20 principal components. In all populations only 1 cluster was identified. Genes which are coding for proteins that are only expressed by a fraction of the cells at protein level, and *CCL5* are shown. **(D,E,F)** To increase the number of cells, our bone marrow NK cells were integrated with single-cell RNA sequencing data from sorted NK cells from the Crinier (8 bone marrow donors, GSE159624) and Yang dataset (two blood and six bone marrow donors, GSE130430). **(D)** The CD56^{bright} NK cells (n=2716), **(E)** CD56^{dim}*GZMK*$^{+}$ NK cells (n=5971) and **(F)** ltNK cells (n=3146) were clustered (k=11) and visualized in a UMAP based on the corrected principal component scores (mutual nearest neighbors correction). In the CD56^{dim}*GZMK*$^{+}$ NK and ltNK cell population multiple clusters were identified. The top 5 most differentially expressed genes for each **(G)** CD56^{dim}*GZMK*$^{+}$ and **(H)** ltNK cell subcluster comparison (either up- or downregulated) were selected. The average scaled expression for each cluster is shown.

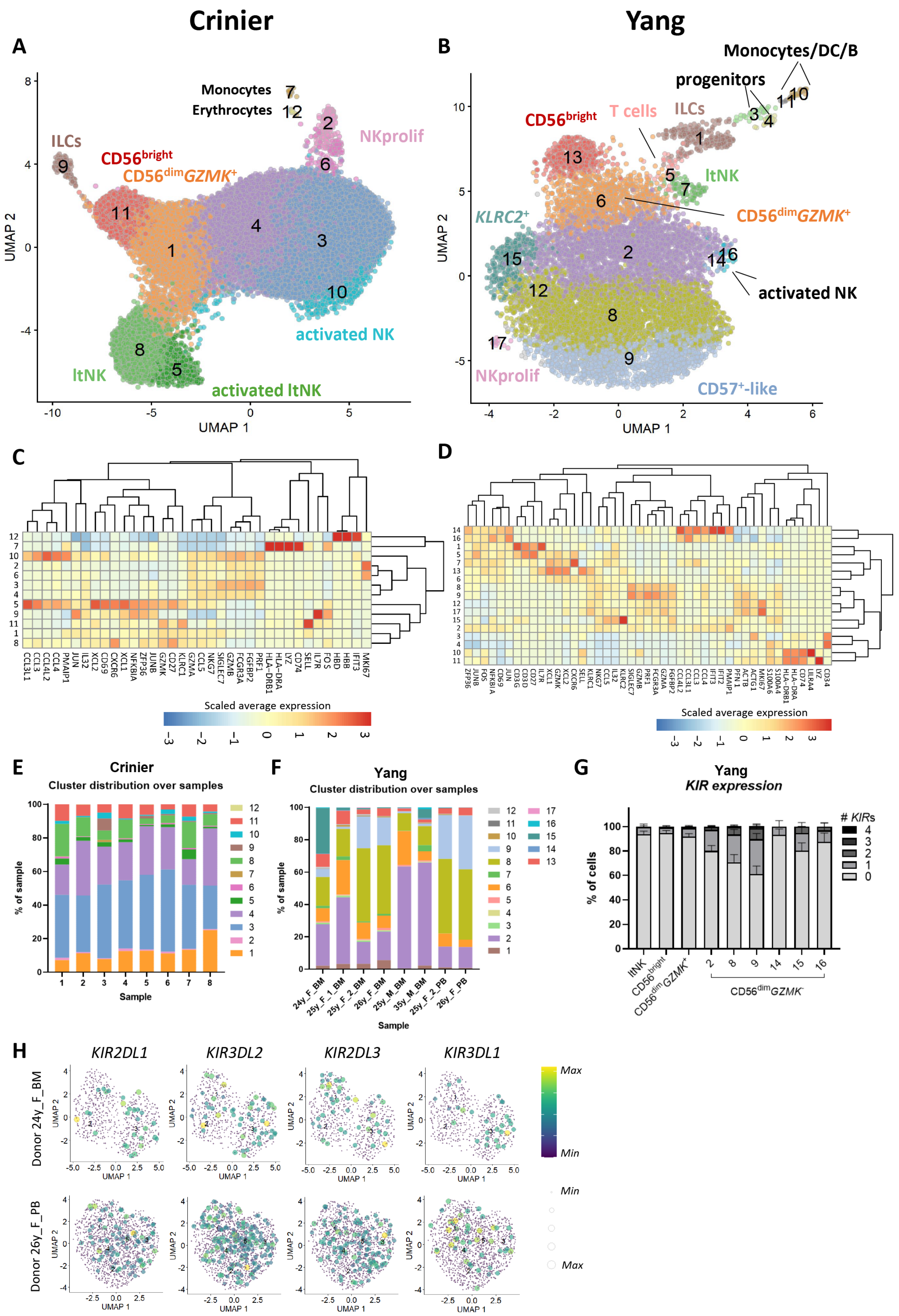
Crinier
Yang
A
B
Monocytes
Erythrocytes
ILCs
CD56bright
CD56dimGZMK+
NKprolif
activated NK
ltNK
activated ltNK
UMAP 1
UMAP 2
Monocytes/DC/B
progenitors
T cells
KLRC2+
CD57+-like
C
D
Scaled average expression
E
Crinier
Cluster distribution over samples
% of sample
Sample
F
Yang
Cluster distribution over samples
G
Yang
KIR expression
% of cells
# KIRs
CD56dimGZMK-
H
KIR2DL1
KIR3DL2
KIR2DL3
KIR3DL1
Donor 24y_F_BM
Donor 26y_F_PB
Max
Min

Figure S3. Analysis of the single-cell RNA sequence dataset from Crinier and Yang et al.
(A) Clustering of the single-cell RNA sequencing dataset (GSE159624) of Crinier et al. containing 24522 cells from 8 bone marrow donors resulted in 12 clusters. ltNK = lymphoid tissue NK. **(B)** Clustering of the single-cell RNA sequencing dataset (GSE130430) of Yang et al. containing 9071 cells from 6 bone marrow and 2 blood donors resulted in 17 clusters. **(C,D)** A selection of genes used to define the clusters of the **C** Crinier and **D** Yang dataset are included in the heatmap. The scaled average expression is shown. **(E,F)** Distribution of clusters per individual sample for the **E** Crinier and **F** Yang dataset. **(G)** The percentage of inhibitory *KIR* expressing cells for each NK cell cluster of the Yang dataset (n=8) was based on *KIR2DL1*, *KIR3DL2*, *KIR2DL3* and *KIR3DL1*. The detection limit for KIR gene expression was set at 0, based on the log2 normalized counts. The proliferating NK cells, representing a mixed population of NK cell subsets, and cluster 12 (<25 cells) were removed. The bars represent mean and standard error of the mean. (**H**) The $CD56^{dim}$*GZMK*$^{-}$ cells from the Yang dataset were selected (cluster 2, 8, 9, 12, 14, 15 and 16) and each dataset was individually clustered. The inhibitory KIR expression of 2 donors with a high percentage of *KLRC2*$^{+}$ and $CD57^{+}$-like NK cells is shown (24y_F_BM and 26y_F_PB, respectively). Again, no inhibitory KIR-driven clustering was observed. Both the color and the size of each dot represent the expression level.

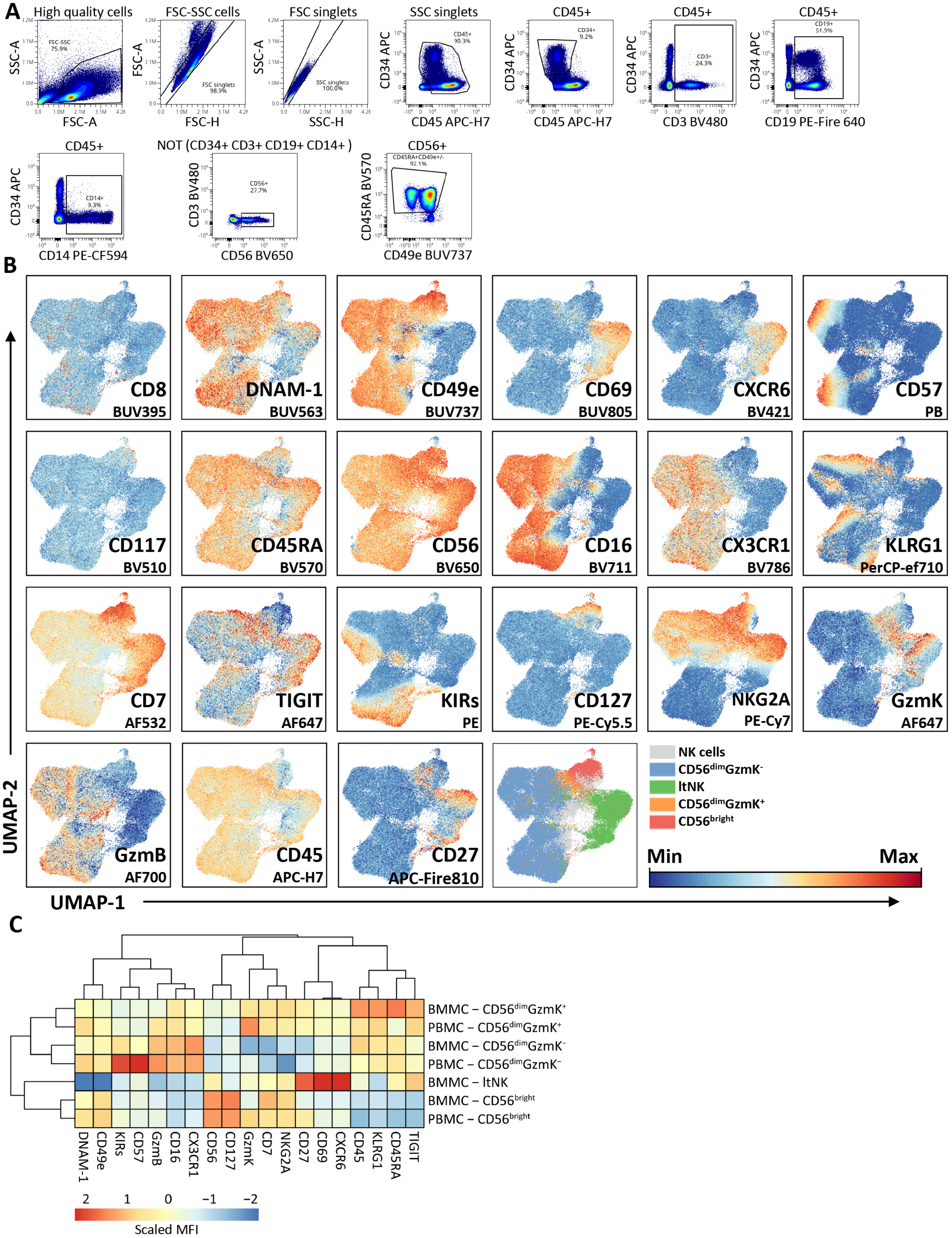
A
High quality cells
SSC-A
FSC-A
FSC-SSC cells
FSC-A
FSC-H
FSC singlets
SSC-A
SSC-H
SSC singlets
CD34 APC
CD45 APC-H7
CD45+
CD34 APC
CD45 APC-H7
CD45+
CD34 APC
CD3 BV480
CD45+
CD34 APC
CD19 PE-Fire 640
CD45+
CD34 APC
CD14 PE-CF594
NOT (CD34+ CD3+ CD19+ CD14+)
CD3 BV480
CD56 BV650
CD56+
CD45RA BV570
CD49e BUV737
B
CD8 BUV395
DNAM-1 BUV563
CD49e BUV737
CD69 BUV805
CXCR6 BV421
CD57 PB
CD117 BV510
CD45RA BV570
CD56 BV650
CD16 BV711
CX3CR1 BV786
KLRG1 PerCP-ef710
CD7 AF532
TIGIT AF647
KIRs PE
CD127 PE-Cy5.5
NKG2A PE-Cy7
GzmK AF647
GzmB AF700
CD45 APC-H7
CD27 APC-Fire810
NK cells
CD56dimGzmK-
ltNK
CD56dimGzmK+
CD56bright
Min
Max
UMAP-2
UMAP-1
C
BMMC – CD56dimGzmK+
PBMC – CD56dimGzmK+
BMMC – CD56dimGzmK-
PBMC – CD56dimGzmK-
BMMC – ltNK
BMMC – CD56bright
PBMC – CD56bright
DNAM-1
CD49e
KIRs
CD57
GzmB
CD16
CX3CR1
CD56
CD127
GzmK
CD7
NKG2A
CD27
CD69
CXCR6
CD45
KLRG1
CD45RA
TIGIT
2
1
0
−1
−2
Scaled MFI

Figure S4. UMAP embedding of spectral cytometry data of 14 bone marrow samples of healthy controls

(A) High quality cells were selected based on the results of FlowAi. Single cells were gated based on forward and side scatter. NK cells were further defined as $CD45^{+}CD34^{-}CD3^{-}CD19^{-}CD14^{-}CD56^{+}CD45RA^{+}$ **(B)** A UMAP embedding of NK cells derived from 14 healthy bone marrow samples, based on 21 NK cell markers as measured by spectral cytometry is shown. Lymphoid tissue (lt)NK cells were defined as $CD69^{+}CD49e^{-}$ (Figure 4A). The non-ltNK cells were subdivided into $CD56^{bright}CD16^{+/-}$ and $CD56^{dim}CD16^{+}$ NK cells. **(C)** The heatmap indicates the scaled mean fluorescence intensity (MFI) expression of NK cell markers on the NK cell subsets in blood (3 donors) and bone marrow (14 donors).

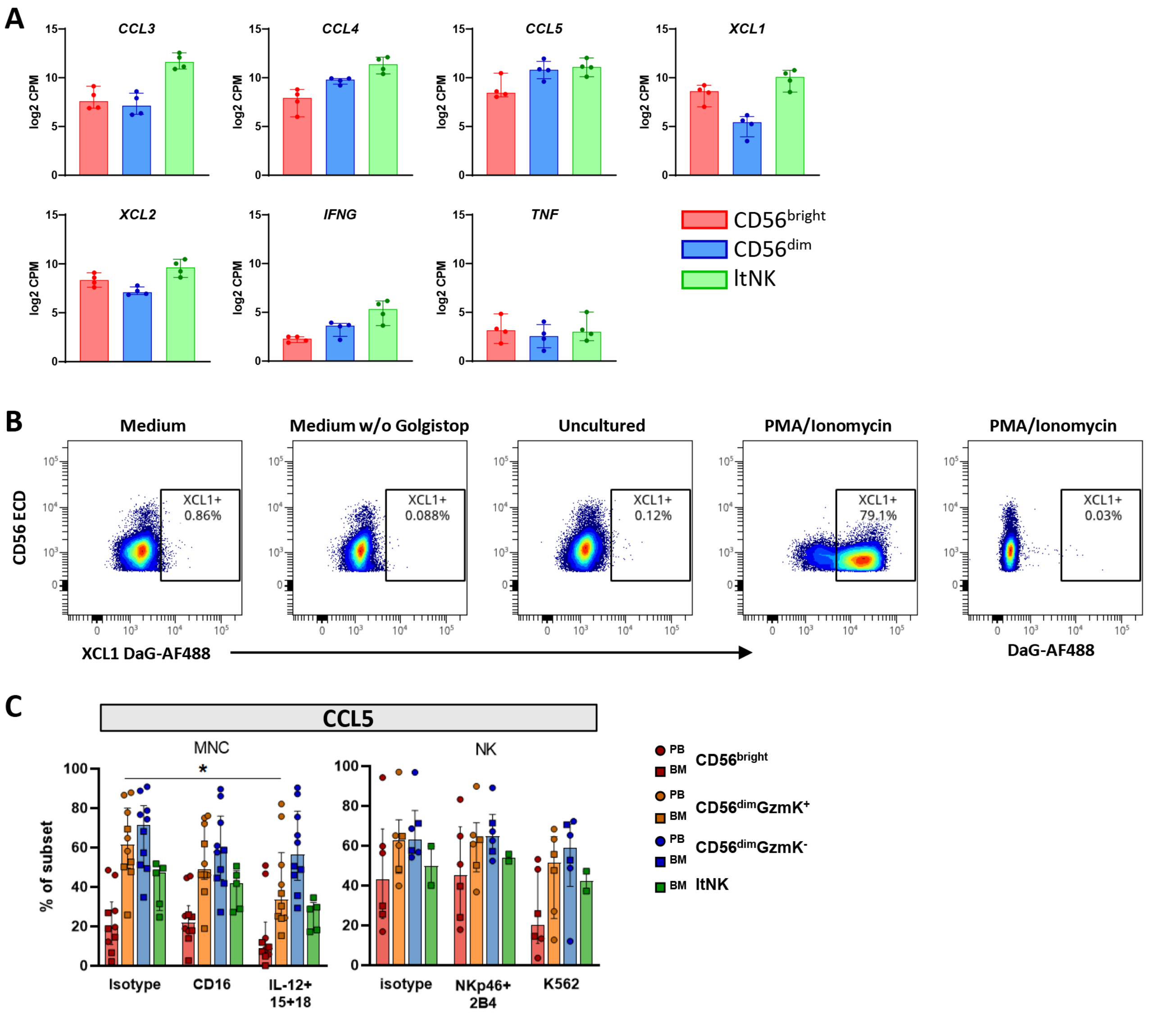

Figure S5. Chemokine and cytokine production of NK cell subsets

(A) Previously published bulk RNA sequence data (GSE116178) showed that each NK cell subset in bone marrow expresses *CCL3*, *CCL4*, *CCL5*, *XCL1*, *XCL2*, *IFNG* and *TNF* at the mRNA level (n=4). Bars indicate median and range. CPM = counts per million reads ltNK = lymphoid tissue NK. **(B)** XCL1 was intracellularly detected by a two-step staining protocol, using unconjugated goat anti-XCL1 and donkey-anti-goat (DaG) AF488. As a negative control, cells were stained with only DaG-AF488 (most right panel). NK cells from a representative blood donor are shown. **(C)** Intracellular CCL5 expression in NK cell subsets, either stimulated in bulk mononuclear cells (MNC, n=5 blood, n=5 bone marrow) or as enriched NK cell fraction (n=4 blood, n=2 bone marrow). Bars indicate median and interquartile range. A paired Friedman test was applied to test for differences with isotype control for each subset. Adjusted P-value*<0.05. PB=peripheral blood, BM=bone marrow.

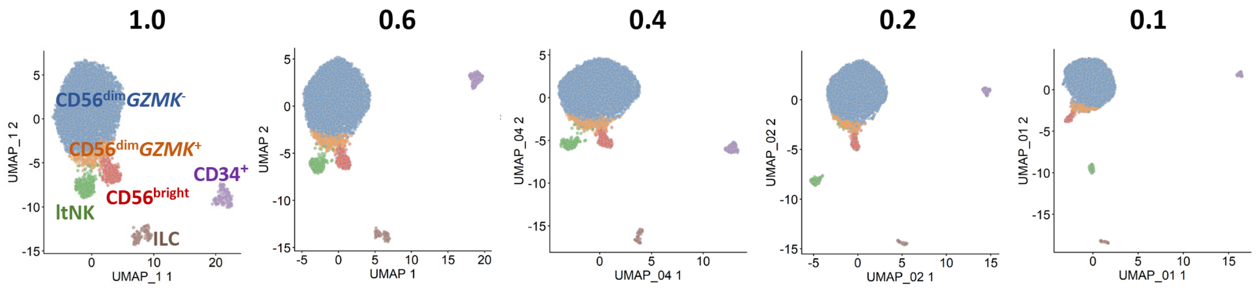

Figure S6. NK cell development

The minimum distance parameter of UMAP (in other UMAP embeddings in this article set at 0.6) was adjusted to study the effect on the embedding. With a lower minimum distance, the CD56^{bright} NK cells were still connected to the CD56^{dim} NK cells, while the lymphoid tissue (lt)NK cells were not.

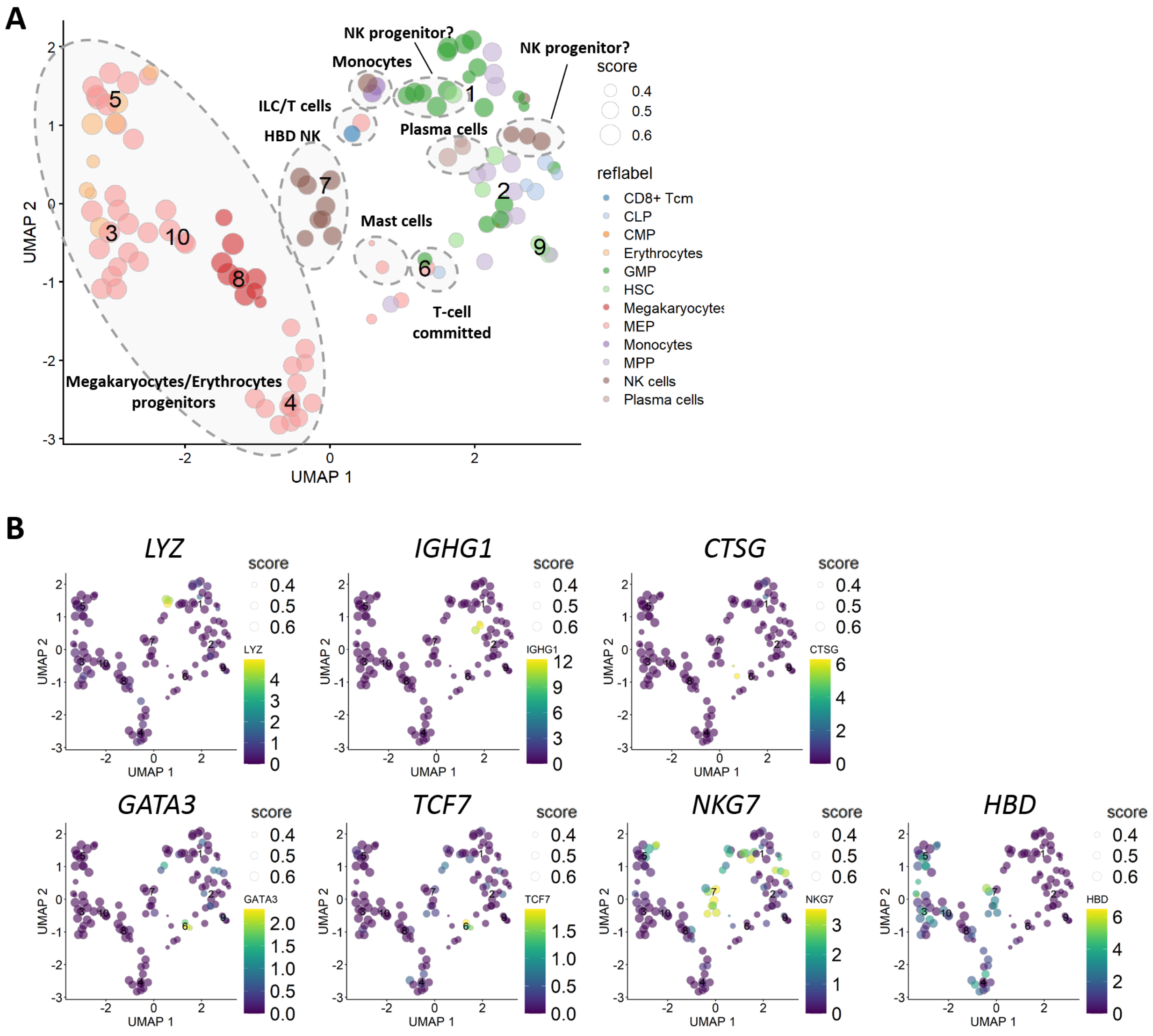

Figure S7. Subclustering of CD34+ population

(A) The CD34+ cells (n=126) in our dataset were selected and subclustered. For the UMAP, n_neighbors=20, and min_dist=0.1 was applied. In addition, reference-based analysis was performed based on bulk RNA sequence data of hematopoietic populations (Blueprint and Encode datasets). The size of the cells reflects the probability score (the bigger, the more likely the assignment of the reference label is correct). The *HBD+NKG7+* cells in cluster 7 are likely to represent NK cells contaminated with lysed erythrocytes. **(B)** The expression of a selection of genes is indicated on the UMAP plot.

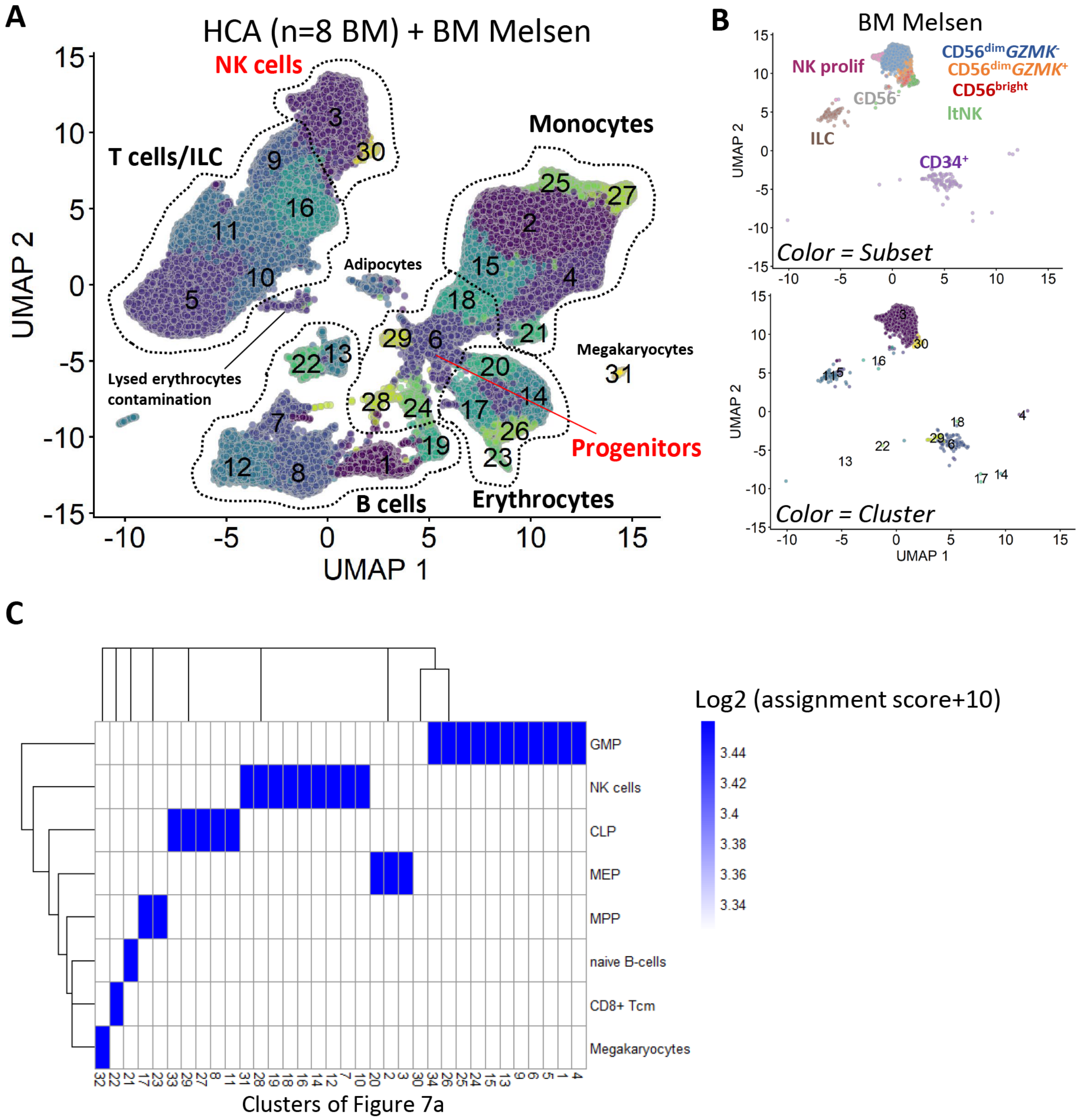

Figure S8. Integration of NK cells with bone marrow cells from the human cell atlas

(A) The bone marrow cells of 8 donors from the human cell atlas (HCA) single-cell RNA sequence dataset were integrated with our bone marrow NK cell dataset (total = 323466 cells). Each color on the UMAP (min. distance=0.4) represents a distinct cluster. **(B)** Cells from our bone marrow NK cell dataset were selected and plotted in the same UMAP embedding as in A. Cells were colored by their original subset assignment, or cluster assignment as indicated in A. ltNK = lymphoid tissue NK **(C)** Cluster 6, 18, 24, 28, 29 (progenitors) and 3, 30 (NK cells) from A were selected and re-clustered (total = 41476 cells), as visualized in Figure 7A. Reference-based analysis using the bulk RNA sequence Blueprint and Encode datasets was performed on each cluster for automatic annotation.

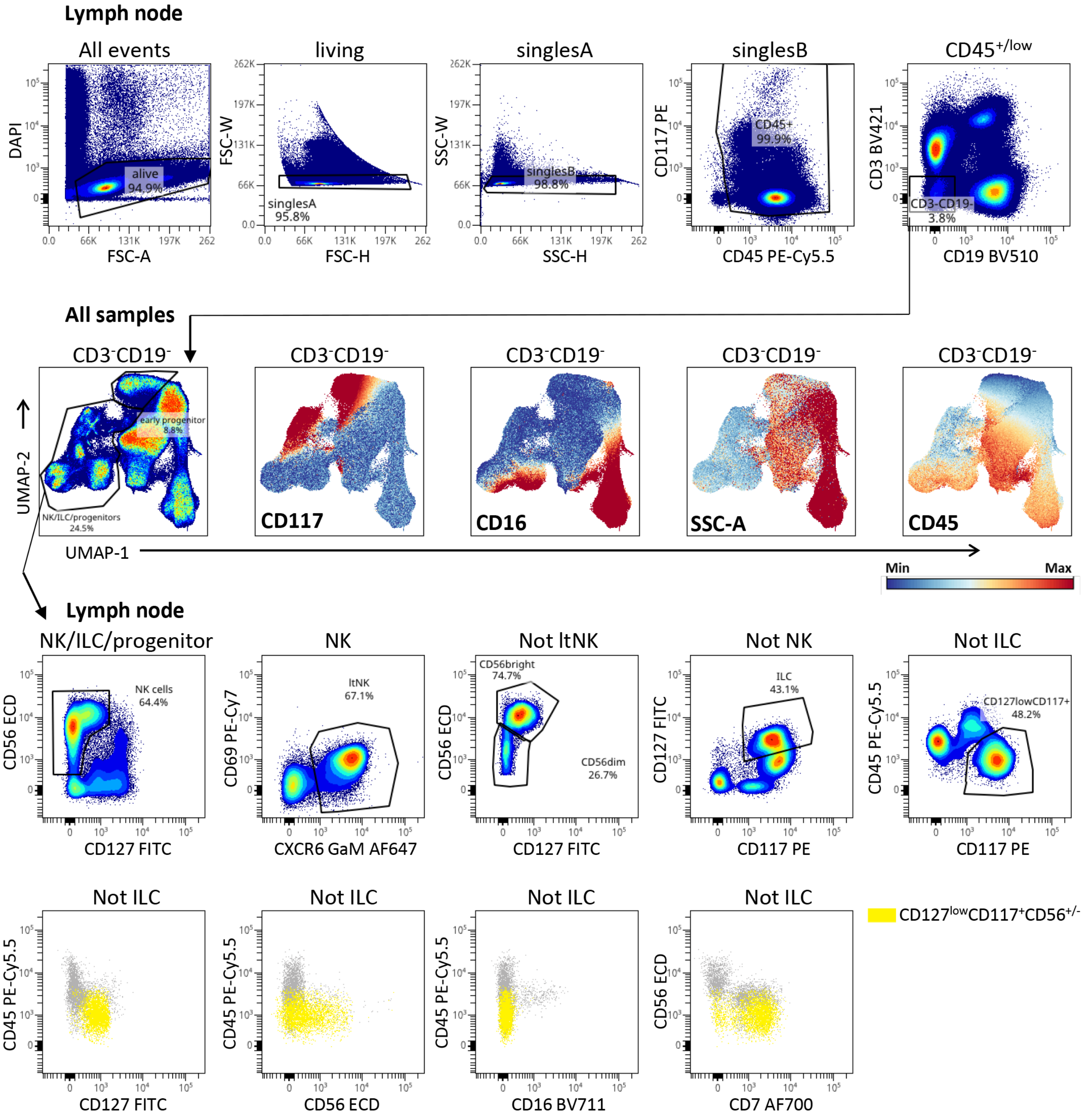

Figure S9. NK cell development in secondary lymphoid tissues

Gating strategy applied to study presence of NK progenitor cells in bone marrow, spleen, lymph node and tonsil. Dead and doublet cells were excluded per individual sample. Next, CD45$^{+/low}$CD3^{-}CD19^{-} cells were gated and all samples were embedded in a UMAP. In the UMAP, early progenitors (CD117^{+}CD16^{-}SSC^{high}CD45^{low}) and a combination of NK, helper innate lymphoid cells (ILCs) and other progenitors were selected. Within the NK/ILC/progenitor gate, NK cell subsets, ILCs and the CD127^{low}CD117^{+}CD56$^{+/-}$ potential NK progenitor cells were defined per individual sample. A representative lymph node donor is shown in the first row, and last two rows. ltNK = lymphoid tissue NK, ILC= innate lymphoid cell.

1.2 Supplementary Tables

Table S1. Antibodies used for flow cytometry

Specificity		Antibody characteristics					
CD designation	Alternative name	Fluorochrome	Type	Clone	Company	Catalog#	Panel
CD3		BV421	m-IgG1	UCHT1	Becton Dickinson	562426	LSR
CD7		AF700	m-IgG1	M-T701	Becton Dickinson	561603	LSR
CD16	FCGR3A	BV711	m-IgG1	3G8	Becton Dickinson	563127	LSR/Aurora
CD19		BV510	m-IgG1	SJ25C1	Becton Dickinson	562947	LSR
CD34		PE	m-IgG1	8G12	Becton Dickinson	345802	LSR
CD45		PE-Cy5.5	m-IgG1	J33	Beckman Coulter	A54139	LSR
CD56	NCAM	APC	m-IgG1	N901	Beckman Coulter	IM2474	LSR
CD56	NCAM	ECD	m-IgG1	N901	Beckman Coulter	A82943	LSR
CD56	NCAM	PE-Cy7	m-IgG1	N901	Beckman Coulter	A21692	LSR
CD69		PE-Cy7	m-IgG1	L78	Becton Dickinson	335792	LSR
CD117	c-KIT	PE	m-IgG1	104D2	Becton Dickinson	332785	LSR
CD117	c-KIT	BV510	m-IgG1	104D2	Biolegend	313220	LSR/Aurora
CD127	IL7R	FITC	m-IgG1	eBioRDR5	eBioscience	11-1278-42	LSR
CD186	CXCR6	Unconjugated	m-IgG2b	56811	R&D	MAB699	LSR
CD186	CXCR6	BV421	m-IgG2a	K041E5	Biolegend	356014	LSR/Aurora
Secondary	m-Ig	APC	g-Ig	polyclonal	Becton Dickinson	550826	LSR
Secondary	m-IgG2b	AF647	g-IgG	polyclonal	Invitrogen	A21242	LSR
CD2		FITC	m-IgG1	MT910	DAKO	f767	Aurora
CD3		BV480	m-IgG1	UCHT1	Becton Dickinson	566105	Aurora
CD3		BV785	m-IgG1	UCHT1	Biolegend	300472	Aurora
CD7		AF532	m-IgG1	eBio124-1D1	eBioscience	58-0079-42	Aurora
CD7		R718	m-IgG1	M-T701	Becton Dickinson	752079	Aurora
CD8		BUV395	m-IgG1	RPA-T8	Becton Dickinson	9317565	Aurora
CD16	FCGR3A	BV480	m-IgG1	3G8	Becton Dickinson	566108	Aurora
CD27		APC-Fire 810	m-IgG1	QA17A18	Biolegend	393213	Aurora
CD45		APC-H7	m-IgG1	2D1	Becton Dickinson	641417	Aurora
CD45		BUV805	m-IgG1	HI30	Becton Dickinson	612891	Aurora
CD45RA		BV570	m-IgG2b	HI100	Biolegend	304132	Aurora
CD49e	ITGA5	BUV737	m-IgG1	IIA1	Becton Dickinson	741849	Aurora
CD49e	ITGA5	BV711	m-IgG1	IIA1	Becton Dickinson	740787	Aurora
CD56	NCAM	BV650	m-IgG2b	NCAM16.2	Becton Dickinson	564057	Aurora
CD57		PB	m-IgM	HNK1	Biolegend	359608	Aurora
CD62L	SELL	BV605	m-IgG1	DREG-56	Biolegend	304834	Aurora
CD69		BUV737	m-IgG1	FN50	Becton Dickinson	612817	Aurora
CD69		BUV805	m-IgG1	FN50	Becton Dickinson	748763	Aurora
CD127	IL7Ra	PE-Cy5.5	m-IgG1	eBioRDR5	eBioscience	35-1278-42	Aurora
CD158a+h	KIR2DL1, KIR2DS1	PE	m-IgG1	EB6	Beckman Coulter	A09778	Aurora
CD158b1+b2+j	KIR2DL2, KIR2DL3, KIR2DS2	PE	m-IgG1	GL183	Beckman Coulter	IM2278U	Aurora
CD158e1	KIR3DL1	PE	m-IgG1	DX9	Becton Dickinson	555967	Aurora
CD158i	KIR2DS4	PE	m-IgG2a	FES172	Beckman Coulter	IM3337	Aurora
CD159a	NKG2A	PE-Cy7	m-IgG2b	Z199	Beckman Coulter	B10246	Aurora
CD226	DNAM-1	BUV563	m-IgG1	DX11	Becton Dickinson	748429	Aurora
CD335	NKp46	Unconjugated	m-IgG1	9E2	Becton Dickinson	557911	Aurora
	CCL4	PerCP-ef710	m-IgG2a	FL34Z3L	eBioscience	46-7540-42	Aurora
	CCL5	PE	m-IgG2b	VL1	Biolegend	515504	Aurora
	CX3CR1	BV786	r-IgG2b	2A9-1	Becton Dickinson	744489	Aurora
	GZMB	AF700	m-IgG1	GB-11	Becton Dickinson	560213	Aurora
	GZMB	PE	m-IgG1	GB-11	Sanquin	M2289	Aurora
	GZMK	AF647	m-IgG1	GM26E7	Biolegend	370503	Aurora
	IFNG	PE-Dazzle594	m-IgG1	4S.B3	Biolegend	502546	Aurora
	KLRG1	PerCP-ef710	m-IgG2a	13F12F2	eBioscience	46-9488-42	Aurora
	TIGIT	AF647	m-IgG1	MBSA43	eBioscience	51-9500-42	Aurora
	TNFa	AF700	m-IgG1	Mab11	Biolegend	502928	Aurora
	XCL1	Unconjugated	g-IgG	polyclonal	R&D	AF695	Aurora
Secondary	g-IgG (H+L)	AF488	d-IgG	polyclonal	Invitrogen	A-11055	Aurora
Secondary	m-IgG1	AF594	g-IgG	polyclonal	Invitrogen	A-21125	Aurora

main abbreviations Type: m=mouse, g=goat, rec=recombinant, h=human, r=rat, g=goat, d=donkey

Table S2. Oligonucleotide-conjugated antibodies used for single-cell RNA sequencing

Specificity		Antibody characteristics				
CD designation	Alternative name	Oligonucleotide	Type	Clone	Company	Catalog#
CD34		GCAGAAATCTCCCTT	m-IgG1	581	Biolegend	343537
CD56	NCAM	TCCTTTCCTGATAGG	m-IgG1	5.1H11	Biolegend	362557
CD117	c-KIT	AGACTAATAGCTGAC	m-IgG1	104D2	Biolegend	313241
CD186	CXCR6	GACAGTCGATGCAAC	m-IgG2a	K041E5	Biolegend	356021

main abbreviations Type: m=mouse
